# The diversity of macromycetes in peatlands: nine years of plot-based monitoring and barcoding in the raised bog "Mukhrino", West Siberia

**DOI:** 10.3897/BDJ.11.e105111

**Published:** 2023-10-20

**Authors:** Nina Filippova, Elena Zvyagina, Elena Rudykina, Alevtina Dobrynina, Sergey Bolshakov

**Affiliations:** 1 Yugra State University, Khanty-Mansiysk, Russia Yugra State University Khanty-Mansiysk Russia; 2 Lomonosov Moscow State University, Moscow, Russia Lomonosov Moscow State University Moscow Russia; 3 Komarov Botanical Institute of the Russian Academy of Sciences, Saint Petersburg, Russia Komarov Botanical Institute of the Russian Academy of Sciences Saint Petersburg Russia

**Keywords:** sphagnum, macromycetes, peat, biodiversity, decomposers, fungal conservation

## Abstract

**Background:**

Peatland ecosystems are defined by soils with sufficient under-decomposed organic layer, called peat, formed under anoxic conditions. Peatlands are widespread around the world, with several highly paludified regions, one of which is the Western Siberian Plain. Peatlands store large amounts of carbon and are important in their intact state to counteract climate change, as well as for a variety of other ecosystem functions. From the practical aspect, these ecosystems are used as a source of peat for fuel, peat-based fertilisers and growing media, berries and *Sphagnum* plantations. Fungi are the key part of the decomposer community of peatlands, playing a critical role in the aerobic decomposition in the upper peat layer. The community of peatland fungi is adapted to decomposition of peat and dead parts of *Sphagnum* in wet acidic conditions; they form specific mycorrhizal associations with a variety of plants. Thus, the research of fungal diversity of peatlands is important for several reasons: 1) adding knowledge of peatland fungal diversity to local or global biodiversity databases; 2) studying carbon cycling in peatlands; 3) using peat and peatlands for different applications, such as cultivation of *Sphagnum* with regards to some parasitic species of fungi and 4) peatland restoration and conservation, to mention a few.

**New information:**

The community of macromycetes of the raised bog “Mukhrino” in Western Siberia was studied using plot-based monitoring throughout a 9-year observation period. The revealed species diversity is represented by approximately 500 specimens in the Fungarium of Yugra State University collection. Selected specimens were used for barcoding of the ITS region to reveal a total of 95 species from 33 genera and three classes. The barcoding effort confirmed morphological identifications for most specimens and identified a number of cryptic species and several potentially new taxa. Based on regular all-season observations, we describe the phenology of the community sporophore production. The quantitative community structure, based on sporophores, revealed a difference in abundance between species by four orders of magnitude, with rare species representing nearly half of the species list. The inter-annual fruiting abundance varied several times by the total number of sporophores per year. To make the comparisons with global studies, we created an open access database of literature-based observations of fungi in peatlands, based on about 120 published papers (comprising about 1300 species) and compared our species list with this database.

As a result, the study created an accurate representation of taxonomic and quantitative structure of the community of macromycetes in raised bogs in the region. The raw data of plot-based counts was published as a sampling-event dataset and the sequenced specimens with the sequence information as an DNA-derived extension dataset in GBIF.

## Introduction

The study of fungal diversity of peatlands started over a century ago with a microbiological approach (for a detailed history of research of fungi in peatlands, see Thormann 2006, Rydin et al. 2006 and Artz 2013. Microfungal communities of peat layer were first studied using *in vitro* culture techniques: fungi were discovered at all depths, but in the lower peat layers, they were considered dormant. The composition and structure of microfungal communities depend on micro-element composition, decomposition state and aerobic conditions of the peat horizons (Thormann 2006, Chernov 2018). The microbiological approach, however, is biased towards species of microfungi easily cultivated *in vitro*. With an integral approach, peatlands became a subject of interest to mycologists working by direct observation of fruit-bodies of macromycetes. Studies of different types of peatlands were carried out in a variety of paludified regions globally; the following works are noteworthy: Favre (1948) Jura mountain range, France; Einhellinger (1976), Einhellinger (1977) Upper Bavaria, Germany; Lange and Lange (1982) Maglemose, northern Denmark; Stasinska (2011) Pomeranian peatlands, Poland; Ohenoja (1974) and Salonen and Saari (1990) Finland; Kalamees (1982), Kalamees and Raitviir (1982) Estonia; Kotlaba (1953), Kotlabam and Kubicka (1960), Vašutová et al. (2021), Vašutová et al. (2023) in Czechia; Chastukhin (1965) north-western Russia. With the recent development of environmental DNA methods of sampling, all methods complement each other, helping to reveal the diversity and ecological role of fungi in peatlands. A number of published works employed the environmental DNA approach in peatlands: Jackson et al. 2008, Elliott et al. 2015, Garcés-Pastor et al. 2019, Kim et al. 2021, Vašutová et al. 2021; however, the results were rarely verified by cultures or direct observation of fruit-bodies.

In this study, we aimed to describe the diversity, community structure and dynamics of macromycetes of peatlands in West Siberia by direct observation and complement it by a molecular approach (barcoding of accumulated collections). In contrast to the existing research, we used permanent plots with regular visits throughout the growing (snow-free) season from May till October and have been monitoring the plots for nine years, which is, to our knowledge, the longest observation series on the community of macromycetes in peatlands. Long-term plot-based monitoring is necessary to reveal the fullest possible diversity of macromycetes, as shown by many authors (O'Dell et al. 2004). There had been no published in-depth studies in regard to macromycetes of peatlands before the beginning of this project in Western Siberia; the study complements several research initiatives on peatland fungal diversity conducted country-wide in Russia. The plot-based description of the community by the direct observation method will be validated or used for validation for future metabarcoding analyses in the same location.

This publication aims:

- to summarise the results of nine years of plot-based monitoring of the community fruiting dynamics;
- to describe the quantitative structure of the community, to reveal dominant and rare species and gain insight into its potential protection status;
- to revise the species diversity of macromycetes by barcoding the voucher collection.

## Materials and methods

### Study site

**Figure 1.**
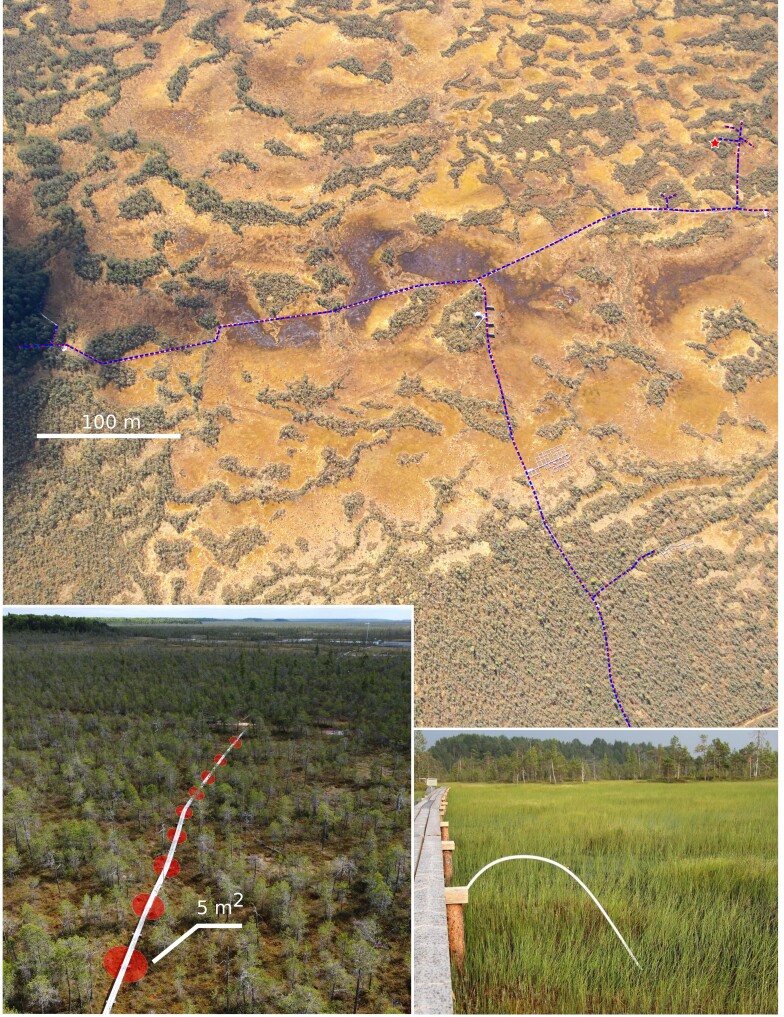
Layout of the Mukhrino field station infrastructure and position of plots (red dots) alongside the walking board (blue dashed line), the star marking the position of an automated meteorological complex; in lower right insert: layout of half-circles on each side of the boardwalk in the treed bog; in lower left insert – counting event in the graminoid-*Sphagnum* bog, using an arch.

The study site – the Mukhrino field station and the Mukhrino Bog – is located in the middle taiga zone of Western Siberia, near the regional capital city of Khanty-Mansiysk (60.89N, 68.68E) (Fig. 1). The Mukhrino Bog is an ombrotrophic landscape entity covering an area of about 10 by 15 km, located along the northern edge of a larger paludified area, the Konda Lowlands (Russian *Кондинская низменность*), on the left terrace of the Irtysh River close to its confluence with the Ob'. The vegetation of the raised bog is represented by the typical ombrotrophic or oligo-mesotrophic communities from the geobotanical classes *Scheuchzerio*-*Cariceteanigrae*, *Oxycocco*-*Sphagnetea* and *Vaccinio*-*Piceetea*. Two major vegetation types dominate: treed Scots pine – dwarf shrubs – *Sphagnum* bogs dominated by *Pinussylvestris*, *Chamaedaphnecalyculata*, *Rhododendrongroenlandicum*, *Rubuschamaemorus* and *Sphagnumfuscum*) and open graminoid-Sphagnum lawns (dominated by *Scheuchzeriapalustris*, *Carexlimosa*, *Eriophorumrusseolum*, *Vacciniumoxycoccos* and *Sphagnumbalticum*).

### Collection of voucher specimens

About 500 specimens were collected during plot-based observations from 2014 through 2022. The specimens are accessible through the Fungarium YSU collection database at the biological portal of the Yugra State University (http://bioportal.ugrasu.ru/; https://fungariumysu.org) or through the collection's dataset in GBIF (Filippova 2023). The collections were processed as described by Lodge and Ammirati (2004). Fresh fruit-bodies were wrapped in aluminium foil and transported to the laboratory to be processed on the day of collection. The processing of specimens included photography in situ and ex situ, description of vital characters, preliminary microscopy and identification, entering new data into the database, labelling and drying at 40°C for packaging and storage in the YSU Fungarium.

For the purpose of barcoding, the collection was revised and specimens of good quality with good photographs and descriptions were selected. Each morphologically-defined taxon was represented by 2-3 specimens on average, with up to 10 specimens for some cryptic taxa.

### Morphological identification

Сommon and easily recognisable species were identified in the field. Thorough identification of collections impossible to identify with certainty in the field was done in the laboratory. Samples from dry specimens were rehydrated in tap water or potassium hydroxide (KOH) (10%), dyes and other chemicals (Congo Red, Melzer's Reagent, ammonia) were applied when necessary. A Zeiss Axiostar microscope with Achromat 5/0.12, 10/0.25, 40/0.65 (dry) and 100/1.25 (oil immersion) objectives were used for microscopical examination. Most of the specimens were identified using Funga Nordica keys (Knudsen and Vesterholt 2018); a number of monographs on particular taxa were used when necessary.

### Molecular methods

We relied primarily on the ITS region using the ITS1-F and ITS4 primers (Gardes and Bruns 1993); for 10 specimens, TEF1 region using the primers EF1-983F and EF1-1567R (Rehner and Buckley 2005) and for three specimens, LSU using the primers LR0R, LR5 (Vilgalys and Hester 1990) were amplified. DNA extraction was done from dry exsiccata or freshly-collected material using Diatom DNA Prep 200 or TransDirect® Plant Tissue PCR Kit following the standard protocols of these kits. The PCR was made using ready mix for PCR 5X ScreenMix (Evrogen). PCR and sequence reaction products were purified using Cleanup Standard (Evrogen), CleanMag DNA (Evrogen) and Dynabeads™ Sequencing Clean-Up kits. Sequencing was performed with BrilliantDye™ Terminator (v.3.1) Cycle Sequencing Kit (NimaGen) using Applied Biosystems® Sanger Sequencing 3500 Series Genetic Analyzer. Sequences were processed with Sequencing Analyst 7 and MEGA 11 software. The resulting sequences were uploaded to GenBank, PlutoF and BOLD (Filippova et al. 2023c) and as a DNA-derived extension dataset to GBIF (Filippova et al. 2023b). The closest or matching taxa were chosen by BLAST at NCBI (https://blast.ncbi.nlm.nih.gov/Blast.cgi) and massBLASTer SH matching (BLAST+ 2.13.0) at PlutoF (https://plutof.ut.ee/en).

The following approach was used to search for the correct taxon name using sequence alignment. The NCBI BLAST search was performed to find the nearest sequences from a type specimen or an authentic specimen with a percentage identity conventionally accepted (for example, 99% for Cortinarius, Liimatainen et al. (2020)). In case no type or authentic specimen exists in NCBI, any other reliable sequence was chosen. The sequences of each group were aligned with the nearest sequence of a type (or an authentic specimen) from this group, trimmed for maximum overlap and the ITS1 and ITS2 regions with the number of nucleotides conventional for this group (including 5.8S) were left. The chosen taxon name with final species assignment, percentage identity and nearest sequences in GenBank are presented in Suppl. material 1. Most of the sequences (107) had 100% similarity and an additional 33 sequences had 99% percentage similarity. However, 15 sequences had much lower threshold (98% and less) and identifications were left at genus level. These specimens should be studied in detail by taxon-specific phylogenetic analyses.

### Statistical analyses

The dataset was analysed and visualised using a collection of packages (Wickham et al. 2019) for R ver. 4.1.2 (RCoreTeam 2021) in RStudio ver. 2021.09.1+372. The «metacoder» package was used to visualise the taxonomic coverage (Foster et al. 2017). The packages «tsibble», «tidyverse», «feasts» and «ggplot2» were used for data analysis and visualisation (Hyndman and Athanasopoulos 2021). The estimation of species diversity was carried out using the «iNEXT.3D» and «iNEXT.4steps» packages (Chao et al. 2020, Chao et al. 2021). The ordination of fungal communities was made using t-SNE ordination (Laurens and Geoffrey 2008).

### Data management and storage

The plot-counts dataset includes two related tables of sampling event format: the basic Event table and the related Occurrence table. The Event table includes 18 fields and about 25 thousand records (263 plots and 96 visits): habitat, geography, date and plot size fields. The Occurrence table includes seven fields and 8284 records of observations with fruit-body counts within each plot on a particular day. The absence of occurrence records corresponding to the record in the Event table denotes an absence of fungi within a plot on a particular day (an approach recommended in GBIF IPT user manual). The database is attached as Suppl. material 2 and can be accessed through GBIF dataset (Filippova et al. 2023a).

Since different number of surveys were carried out in different years, we used a standardisation procedure before any comparative analyses could be performed. We transformed the actual data to a matrix of 9 years per 13 decade counts, where each year had an equal number of counts from the end of May to the end of September. Empty decades (without a single count) were interpolated by calculating the mean value between adjacent counts.

Since many morphological species were re-identified into several taxa, based on molecular analysis, we used composite names for these species (e.g. *Cortinariusarmeniacus*/*kauffmanianus* or adding *s.l.* in some cases).

The sequenced specimens dataset includes two connected tables: the occurrence table and the DNA-derived data extension table (https://rs.gbif.org/extension/gbif/1.0/dna_derived_data_2021-07-05.xml#DNA_sequence). The Occurrence table contains the descriptions (in 22 fields) of 149 sequenced specimens, including images of fresh fruit-bodies. The related DNA-derived data extension table provides 155 sequences with descriptions of their parameters in eight fields. The dataset can be accessed through GBIF (Filippova et al. 2023b).

The literature-based dataset contains roughly 5000 citations of data on peatland fungi globally. Additional information includes bibliographic citation, date, country where the species was studied, habitat and substrate. The database can be accessed through GBIF (Filippova and Rudykina 2023). To avoid a mix-up of taxonomical concepts in literature datasets, names were synonymised using the GBIF species matching tool following the GBIF Backbone taxonomy.

## Checklists

### The checklist of macrofungi collected during the monitoring in raised bogs with associated sequences

#### Amanita porphyria

Alb. & Schwein.

16DBBCF6-3A18-5821-B20B-A9F4F643C7F1

##### Materials

- **Type status:** Other material. **Occurrence:** catalogNumber: YSU-F-04416; recordedBy: Filippova, Nina; associatedSequences: OP866197; occurrenceID: A7AEF718-99A1-509F-9458-802CCF98FFF5; **Location:** country: Russian Federation; countryCode: RU; stateProvince: Khanty-Mansiyskiy Avtonomnyy Okrug; county: Khanty-Mansiyskiy Rayon; locality: Mukhrino field station of YSU, 20 km SW from Khanty-Mansiysk; decimalLatitude: 60.892632; decimalLongitude: 68.677156; **Identification:** identifiedBy: Filippova, Nina|Zvyagina, Elena; dateIdentified: 2023-02-28; identificationRemarks: Identification based on morphological and molecular characters; **Event:** eventDate: 2013-09-07; habitat: Dwarfshrubs - sphagnum ombrotrophic bog

#### Arrhenia bigelowii

Voitk, Lickey & I.Saar, 2022

F0BC6B82-A0F3-548D-A8ED-554C437F4FBD

##### Materials

- **Type status:** Other material. **Occurrence:** catalogNumber: YSU-F-12021; recordedBy: Filippova, Nina; associatedSequences: OP866238; occurrenceID: 6B8C0121-5C1A-509E-81F1-BA80CE006001; **Location:** country: Russian Federation; countryCode: RU; stateProvince: Khanty-Mansiyskiy Avtonomnyy Okrug; county: Khanty-Mansiyskiy Rayon; locality: Mukhrino field station of YSU, 20 km SW from Khanty-Mansiysk; decimalLatitude: 60.891781; decimalLongitude: 68.684251; **Identification:** identifiedBy: Filippova, Nina|Zvyagina, Elena; dateIdentified: 2023-02-28; identificationRemarks: Identification based on morphological and molecular characters; **Event:** eventDate: 2022-07-11; habitat: Raised Sphagnum bog
- **Type status:** Other material. **Occurrence:** catalogNumber: YSU-F-12125; recordedBy: Filippova, Nina|Rudykina, Elena; associatedSequences: OP866246; occurrenceID: 5DBDC9C1-A067-5903-85A9-A3F37609D77D; **Location:** country: Russian Federation; countryCode: RU; stateProvince: Khanty-Mansiyskiy Avtonomnyy Okrug; county: Khanty-Mansiyskiy Rayon; locality: Mukhrino field station of YSU, 20 km SW from Khanty-Mansiysk; decimalLatitude: 60.891781; decimalLongitude: 68.684251; **Identification:** identifiedBy: Filippova, Nina|Zvyagina, Elena; dateIdentified: 2023-02-28; identificationRemarks: Identification based on morphological and molecular characters; **Event:** eventDate: 2022-08-04; habitat: Raised Sphagnum bog
- **Type status:** Other material. **Occurrence:** catalogNumber: YSU-F-08245; recordedBy: Filippova, Nina; associatedSequences: OP866223; occurrenceID: 8D1F2D46-306E-59DF-B0CB-60EFC3D68D45; **Location:** country: Russian Federation; countryCode: RU; stateProvince: Khanty-Mansiyskiy Avtonomnyy Okrug; county: Khanty-Mansiyskiy Rayon; locality: Mukhrino field station of YSU, 20 km SW from Khanty-Mansiysk; decimalLatitude: 60.891781; decimalLongitude: 68.684251; **Identification:** identifiedBy: Filippova, Nina|Zvyagina, Elena; dateIdentified: 2023-02-28; identificationRemarks: Identification based on morphological and molecular characters; **Event:** eventDate: 2018-08-04; habitat: Raised Sphagnum bog

#### Arrhenia gerardiana

(Peck) Elborne

9CA4F2E7-8EA7-5068-94BD-DC6D456B0FC1

##### Materials

- **Type status:** Other material. **Occurrence:** catalogNumber: YSU-F-08514; recordedBy: Filippova, Nina; associatedSequences: OP866228; occurrenceID: 1C2FC960-006E-51BE-94C0-01B17E37D787; **Location:** country: Russian Federation; countryCode: RU; stateProvince: Tomskaya Oblast'; county: Tomskiy Rayon; locality: Orlovka village vicinity, Chernoye lake; decimalLatitude: 56.878320; decimalLongitude: 84.665770; **Identification:** identifiedBy: Filippova, Nina|Zvyagina, Elena; dateIdentified: 2023-02-28; identificationRemarks: Identification based on morphological and molecular characters; **Event:** eventDate: 2018-08-22; habitat: Raised Pine-dwarfshrubs-Sphagnum bog
- **Type status:** Other material. **Occurrence:** catalogNumber: YSU-F-08855; recordedBy: Filippova, Nina; associatedSequences: OP866230; occurrenceID: 0DACEE8B-D278-5278-BF5D-505F90290B39; **Location:** country: Russian Federation; countryCode: RU; stateProvince: Khanty-Mansiyskiy Avtonomnyy Okrug; county: Khanty-Mansiyskiy Rayon; locality: Chistoe bog, 20 km E from Khanty-Mansiysk; decimalLatitude: 61.046340; decimalLongitude: 69.440660; **Identification:** identifiedBy: Filippova, Nina|Zvyagina, Elena; dateIdentified: 2023-02-28; identificationRemarks: Identification based on morphological and molecular characters; **Event:** eventDate: 2019-07-06; habitat: Raised Sphagnum bog

#### Arrhenia philonotis

(Lasch) Redhead, Lutzoni, Moncalvo & Vilgalys

60446006-2947-587E-9A1D-481BB9F24A7D

##### Materials

- **Type status:** Other material. **Occurrence:** catalogNumber: YSU-F-08140; recordedBy: Filippova, Nina; associatedSequences: OP866221; occurrenceID: DC4AF85A-9FF4-58AB-BF01-39563377BDF3; **Location:** country: Russian Federation; countryCode: RU; stateProvince: Khanty-Mansiyskiy Avtonomnyy Okrug; county: Sovetskiy Rayon; locality: Potanay oilfield area; decimalLatitude: 61.188503; decimalLongitude: 65.456627; **Identification:** identifiedBy: Filippova, Nina|Zvyagina, Elena; dateIdentified: 2023-02-28; identificationRemarks: Identification based on morphological and molecular characters; **Event:** eventDate: 2018-07-29; habitat: Raised Sphagnum bog

#### Arrhenia sp.

40CA7486-3E1E-56DF-AE93-6A7AAE662B81

##### Materials

- **Type status:** Other material. **Occurrence:** catalogNumber: YSU-F-08148; recordedBy: Filippova, Nina; associatedSequences: OQ396707; occurrenceID: 033E8211-350D-5E1E-A764-83B8BD5FAB27; **Location:** country: Russian Federation; countryCode: RU; stateProvince: Khanty-Mansiyskiy Avtonomnyy Okrug; county: Sovetskiy Rayon; locality: Potanay oilfield area; decimalLatitude: 61.188503; decimalLongitude: 65.456627; **Identification:** identifiedBy: Filippova, Nina|Zvyagina, Elena; dateIdentified: 2023-02-28; identificationRemarks: Identification based on morphological and molecular characters; **Event:** eventDate: 2018-07-29; habitat: Raised Sphagnum bog

#### Ascocoryne turficola

(Boud.) Korf

442C7A61-DA89-5BF1-B3E5-32A9D9124595

##### Materials

- **Type status:** Other material. **Occurrence:** catalogNumber: YSU-F-06580; recordedBy: Zvyagina, Elena|Zvyagina, Elena; associatedSequences: OP866214; occurrenceID: FFE85935-0D44-5D1D-BC12-8F99EFDE0EBD; **Location:** country: Russian Federation; countryCode: RU; stateProvince: Khanty-Mansiyskiy Avtonomnyy Okrug; county: Surgutskiy Rayon; locality: Yuganskiy State Nature Reserve; decimalLatitude: 60.021295; decimalLongitude: 74.462242; **Identification:** identifiedBy: Filippova, Nina|Zvyagina, Elena; dateIdentified: 2023-02-28; identificationRemarks: Identification based on morphological and molecular characters; **Event:** eventDate: 2016-06-29; habitat: The community of dwarf shrubs and sphagnum, with micro complexity of hummocks and hollows
- **Type status:** Other material. **Occurrence:** catalogNumber: YSU-F-12193; recordedBy: Filippova, Nina|Rudykina, Elena; occurrenceID: 1BDD84B8-7D0F-52CB-9BAA-74F47E4323E4; **Location:** country: Russian Federation; countryCode: RU; stateProvince: Khanty-Mansiyskiy Avtonomnyy Okrug; county: Khanty-Mansiyskiy Rayon; locality: Mukhrino FS boardwalks, 20 km SW from Khanty-Mansiysk; decimalLatitude: 60.891781; decimalLongitude: 68.684251

#### Auriscalpium vulgare

Gray

49991A0C-1AB9-57C4-A894-71A40235B9A1

##### Materials

- **Type status:** Other material. **Occurrence:** recordedBy: Filippova, Nina; occurrenceID: DE2249A1-483A-5CF5-9C3C-2F1AE15F0B67; **Location:** country: Russian Federation; countryCode: RU; county: Khanty-Mansiyskiy Rayon; locality: Mukhrino field station of YSU, 20 km SW from Khanty-Mansiysk; decimalLatitude: 60.891781; decimalLongitude: 68.684251; **Identification:** identifiedBy: Filippova, Nina; identificationRemarks: Identification based on observation, no collections were made; **Event:** eventDate: 2022-08-19; habitat: Raised Sphagnum bog

#### Cantharellula umbonata

(J.F.Gmel.) Singer

B3AC4C14-C673-5485-BFD6-16781059D38F

##### Materials

- **Type status:** Other material. **Occurrence:** catalogNumber: YSU-F-04503; recordedBy: Filippova, Nina; occurrenceID: 388596C9-3E8A-5BFA-A3A0-8591D4CCE254; **Location:** country: Russian Federation; countryCode: RU; county: Khanty-Mansiyskiy Rayon; locality: Mukhrino field station of YSU, 20 km SW from Khanty-Mansiysk; decimalLatitude: 60.89; decimalLongitude: 68.67; **Identification:** identifiedBy: Filippova, Nina; dateIdentified: 2014-08-09; identificationRemarks: Identification based on morphological characters only; **Event:** eventDate: 2014-08-09; habitat: Pine - dwarfshrubs - sphagnum ombrotrophic bog

#### Clavaria sphagnicola

Boud.

CAA8679E-E4B6-5EF9-9F2D-734F76F97004

##### Materials

- **Type status:** Other material. **Occurrence:** catalogNumber: YSU-F-05830; recordedBy: Filippova, Nina; occurrenceID: DD58AF74-84E8-54B3-A582-87DB1A99DB61; **Location:** country: Russian Federation; countryCode: RU; county: Khanty-Mansiyskiy Rayon; locality: Mukhrino field station of YSU, 20 km SW from Khanty-Mansiysk; decimalLatitude: 60.891781; decimalLongitude: 68.684251; **Identification:** identifiedBy: Filippova, Nina; dateIdentified: 2014-08-08; identificationRemarks: Identification based on morphological characters only; **Event:** eventDate: 2014-08-08; habitat: Pine - dwarfshrubs - S. fuscum ombrotrophic bog

#### Collybia cirrhata

(Schumach.) Quél.

EEF4582D-A62D-51B7-8891-F8A247C22830

##### Materials

- **Type status:** Other material. **Occurrence:** catalogNumber: YSU-F-04415; recordedBy: Filippova, Nina; occurrenceID: 31D64D45-463D-5490-B638-220C25356110; **Location:** country: Russian Federation; countryCode: RU; county: Khanty-Mansiyskiy Rayon; locality: Mukhrino field station of YSU, 20 km SW from Khanty-Mansiysk; decimalLatitude: 60.892632; decimalLongitude: 68.677156; **Identification:** identifiedBy: Filippova, Nina; dateIdentified: 2013-09-05; identificationRemarks: Identification based on morphological characters only; **Event:** eventDate: 2013-09-05; habitat: Dwarfshrubs - sphagnum ombrotrophic bog

#### Cortinarius armeniacus

(Schaeff.) Fr.

9EE54258-083F-56D1-B68C-D0505D9FDB8F

##### Materials

- **Type status:** Other material. **Occurrence:** catalogNumber: YSU-F-06277; recordedBy: Filippova, Nina; associatedSequences: OQ366589; occurrenceID: 09FD8B86-1C37-505D-A9DA-4192E5AB613F; **Location:** country: Russian Federation; countryCode: RU; stateProvince: Khanty-Mansiyskiy Avtonomnyy Okrug; county: Khanty-Mansiyskiy Rayon; locality: Mukhrino field station of YSU, 20 km SW from Khanty-Mansiysk; decimalLatitude: 60.892010; decimalLongitude: 68.682420; **Identification:** identifiedBy: Filippova, Nina|Zvyagina, Elena; dateIdentified: 2023-02-28; identificationRemarks: Identification based on morphological and molecular characters; **Event:** eventDate: 2015-08-24; habitat: Pine - dwarfshrubs - S. fuscum ombrotrophic bog
- **Type status:** Other material. **Occurrence:** catalogNumber: YSU-F-06278; recordedBy: Filippova, Nina; associatedSequences: OP866211; occurrenceID: A1F0E284-E221-5DFA-9D74-A0BD66CAD870; **Location:** country: Russian Federation; countryCode: RU; stateProvince: Khanty-Mansiyskiy Avtonomnyy Okrug; county: Khanty-Mansiyskiy Rayon; locality: Mukhrino field station of YSU, 20 km SW from Khanty-Mansiysk; decimalLatitude: 60.892010; decimalLongitude: 68.682420; **Identification:** identifiedBy: Filippova, Nina|Zvyagina, Elena; dateIdentified: 2023-02-28; identificationRemarks: Identification based on morphological and molecular characters; **Event:** eventDate: 2015-08-24; habitat: Pine - dwarfshrubs - S. fuscum ombrotrophic bog
- **Type status:** Other material. **Occurrence:** catalogNumber: YSU-F-03969; recordedBy: Filippova, Nina; associatedSequences: OP866185; occurrenceID: 52F90AE8-9C78-510A-834C-3E56257A9D81; **Location:** country: Russian Federation; countryCode: RU; stateProvince: Khanty-Mansiyskiy Avtonomnyy Okrug; county: Khanty-Mansiyskiy Rayon; locality: Mukhrino field station of YSU, 20 km SW from Khanty-Mansiysk; decimalLatitude: 60.889934; decimalLongitude: 68.700686; **Identification:** identifiedBy: Filippova, Nina|Zvyagina, Elena; dateIdentified: 2023-02-28; identificationRemarks: Identification based on morphological and molecular characters; **Event:** eventDate: 2012-09-02; habitat: Pine - dwarfshrubs - sphagnum bog (close to forest)
- **Type status:** Other material. **Occurrence:** catalogNumber: YSU-F-07410; recordedBy: Filippova, Nina; associatedSequences: OP866219; occurrenceID: CB61994E-1632-5D1B-9D3D-8C31D0DA5365; **Location:** country: Russian Federation; countryCode: RU; stateProvince: Khanty-Mansiyskiy Avtonomnyy Okrug; county: Khanty-Mansiyskiy Rayon; locality: Mukhrino field station of YSU, 20 km SW from Khanty-Mansiysk; decimalLatitude: 60.891781; decimalLongitude: 68.684251; **Identification:** identifiedBy: Filippova, Nina|Zvyagina, Elena; dateIdentified: 2023-02-28; identificationRemarks: Identification based on morphological and molecular characters; **Event:** eventDate: 2016-09-13; habitat: Pine-dwarfshrubs-S.fuscum ombrotrophic bog
- **Type status:** Other material. **Occurrence:** catalogNumber: YSU-F-10526; recordedBy: Filippova, Nina; associatedSequences: OP866233; occurrenceID: 59C9DCC7-CB1A-5BFE-8B1E-4B90ACA95F08; **Location:** country: Russian Federation; countryCode: RU; stateProvince: Khanty-Mansiyskiy Avtonomnyy Okrug; county: Khanty-Mansiyskiy Rayon; locality: Khanty-Mansiysk town vicinity; decimalLatitude: 60.891900; decimalLongitude: 68.682260; **Identification:** identifiedBy: Filippova, Nina|Zvyagina, Elena; dateIdentified: 2023-02-28; identificationRemarks: Identification based on morphological and molecular characters; **Event:** eventDate: 2020-09-08; habitat: Raised Sphagnum bog

#### Cortinarius aurantiobasis

Ammirati & A.H.Sm.

1F09C1FB-9210-5A2D-A8A0-81658A79DC89

##### Materials

- **Type status:** Other material. **Occurrence:** catalogNumber: YSU-F-05842; recordedBy: Filippova, Nina; associatedSequences: OP866208; occurrenceID: 7FC77C06-4EA5-5B32-84A6-7B50F58B0ED8; **Location:** country: Russian Federation; countryCode: RU; stateProvince: Khanty-Mansiyskiy Avtonomnyy Okrug; county: Khanty-Mansiyskiy Rayon; locality: Mukhrino field station of YSU, 20 km SW from Khanty-Mansiysk; decimalLatitude: 60.891781; decimalLongitude: 68.684251; **Identification:** identifiedBy: Filippova, Nina|Zvyagina, Elena; dateIdentified: 2023-02-28; identificationRemarks: Identification based on morphological and molecular characters; **Event:** eventDate: 2015-08-08; habitat: Pine - dwarfshrubs - S. fuscum ombrotrophic bog

#### Cortinarius bataillei

(J.Favre ex M.M.Moser) Høil.

5187FC35-1EBF-56A0-B9A0-6AC980D5CC58

##### Materials

- **Type status:** Other material. **Occurrence:** catalogNumber: YSU-F-04090; recordedBy: Filippova, Nina; associatedSequences: OP866190; occurrenceID: 7266D007-4149-53E0-86D6-C0FA1F1B9918; **Location:** country: Russian Federation; countryCode: RU; stateProvince: Khanty-Mansiyskiy Avtonomnyy Okrug; county: Khanty-Mansiyskiy Rayon; locality: Mukhrino field station of YSU, 20 km SW from Khanty-Mansiysk; decimalLatitude: 60.892022; decimalLongitude: 68.691502; **Identification:** identifiedBy: Filippova, Nina|Zvyagina, Elena; dateIdentified: 2023-02-28; identificationRemarks: Identification based on morphological and molecular characters; **Event:** eventDate: 2012-09-09; habitat: Pine - dwarfshrubs - sphagnum ombrotrophic bog
- **Type status:** Other material. **Occurrence:** catalogNumber: YSU-F-12164; recordedBy: Rudykina, Elena|Dobrynina, Alevtina; associatedSequences: OP866252; occurrenceID: E57AD120-5B3C-5B46-A616-A42A2E700608; **Location:** country: Russian Federation; countryCode: RU; stateProvince: Khanty-Mansiyskiy Avtonomnyy Okrug; county: Khanty-Mansiyskiy Rayon; locality: Mukhrino field station of YSU, 20 km SW from Khanty-Mansiysk; decimalLatitude: 60.891781; decimalLongitude: 68.684251; **Identification:** identifiedBy: Filippova, Nina|Zvyagina, Elena; dateIdentified: 2023-02-28; identificationRemarks: Identification based on morphological and molecular characters; **Event:** eventDate: 2022-08-19; habitat: Raised Sphagnum bog

#### Cortinarius brunneotinctus

Niskanen, Liimat., Ammirati, André Paul & Lebeuf

DDC3E0EB-4C10-5240-AA39-AF86A51C7CBE

##### Materials

- **Type status:** Other material. **Occurrence:** catalogNumber: YSU-F-07111; recordedBy: Filippova, Nina; associatedSequences: OQ366575; occurrenceID: 98747B4E-3D95-52E6-85A1-053C60C0D754; **Location:** country: Russian Federation; countryCode: RU; stateProvince: Khanty-Mansiyskiy Avtonomnyy Okrug; county: Khanty-Mansiyskiy Rayon; locality: Mukhrino field station of YSU, 20 km SW from Khanty-Mansiysk; decimalLatitude: 60.891781; decimalLongitude: 68.684251; **Identification:** identifiedBy: Filippova, Nina|Zvyagina, Elena; dateIdentified: 2023-02-28; identificationRemarks: Identification based on morphological and molecular characters; **Event:** eventDate: 2016-08-13; habitat: Pine-dwarfshrubs-S.fuscum ombrotrophic bog

#### Thaxterogaster causticus

Fr.

9E85D5FD-4BA3-558A-84D6-C3B82DA69EF6

##### Materials

- **Type status:** Other material. **Occurrence:** catalogNumber: YSU-F-04409; recordedBy: Filippova, Nina; associatedSequences: OP866196; occurrenceID: C373D94A-B2E6-5829-A84C-92CC3054CE3F; **Location:** country: Russian Federation; countryCode: RU; stateProvince: Khanty-Mansiyskiy Avtonomnyy Okrug; county: Khanty-Mansiyskiy Rayon; locality: Mukhrino field station of YSU, 20 km SW from Khanty-Mansiysk; decimalLatitude: 60.892632; decimalLongitude: 68.677156; **Identification:** identifiedBy: Filippova, Nina|Zvyagina, Elena; dateIdentified: 2023-02-28; identificationRemarks: Identification based on morphological and molecular characters; **Event:** eventDate: 2013-09-02; habitat: Dwarfshrubs - sphagnum ombrotrophic bog
- **Type status:** Other material. **Occurrence:** catalogNumber: YSU-F-03944; recordedBy: Filippova, Nina; associatedSequences: OQ366579; occurrenceID: ADE1FB6C-6C0D-5C4E-ABB5-8591037917AD; **Location:** country: Russian Federation; countryCode: RU; stateProvince: Khanty-Mansiyskiy Avtonomnyy Okrug; county: Khanty-Mansiyskiy Rayon; locality: Mukhrino field station of YSU, 20 km SW from Khanty-Mansiysk; decimalLatitude: 60.889934; decimalLongitude: 68.700686; **Identification:** identifiedBy: Filippova, Nina|Zvyagina, Elena; dateIdentified: 2023-02-28; identificationRemarks: Identification based on morphological and molecular characters; **Event:** eventDate: 2012-09-01; habitat: Pine - dwarfshrubs - sphagnum bog (close to forest)

#### Cortinarius cinnamomeus

(L.) Gray

D45A910C-CA79-5932-8B2B-5702CB8ABBBB

##### Materials

- **Type status:** Other material. **Occurrence:** catalogNumber: YSU-F-12166; recordedBy: Rudykina, Elena|Dobrynina, Alevtina; associatedSequences: OP866254; occurrenceID: 76D43F18-8811-571D-A13C-39BD1AF5DAB1; **Location:** country: Russian Federation; countryCode: RU; stateProvince: Khanty-Mansiyskiy Avtonomnyy Okrug; county: Khanty-Mansiyskiy Rayon; locality: Mukhrino field station of YSU, 20 km SW from Khanty-Mansiysk; decimalLatitude: 60.891781; decimalLongitude: 68.684251; **Identification:** identifiedBy: Filippova, Nina|Zvyagina, Elena; dateIdentified: 2023-02-28; identificationRemarks: Identification based on morphological and molecular characters; **Event:** eventDate: 2022-08-19; habitat: Raised Sphagnum bog

#### Cortinarius coleoptera

H.Lindstr. & Soop

D61E29C0-6AA6-5010-9059-D9A7A6EBAA6A

##### Materials

- **Type status:** Other material. **Occurrence:** catalogNumber: YSU-F-08357; recordedBy: Filippova, Nina; associatedSequences: OP866224; occurrenceID: F94994DD-6483-5139-8620-7C4165C7D5AE; **Location:** country: Russian Federation; countryCode: RU; stateProvince: Khanty-Mansiyskiy Avtonomnyy Okrug; county: Khanty-Mansiyskiy Rayon; locality: Mukhrino field station of YSU, 20 km SW from Khanty-Mansiysk; decimalLatitude: 60.891781; decimalLongitude: 68.684251; **Identification:** identifiedBy: Filippova, Nina|Zvyagina, Elena; dateIdentified: 2023-02-28; identificationRemarks: Identification based on morphological and molecular characters; **Event:** eventDate: 2018-08-28; habitat: Treed Pine-dwarfshrubs-Sphagnum bog
- **Type status:** Other material. **Occurrence:** catalogNumber: YSU-F-12168; recordedBy: Rudykina, Elena|Dobrynina, Alevtina; associatedSequences: OP866256; occurrenceID: E8F425CA-0F70-52E2-AD68-C404F9B55F8A; **Location:** country: Russian Federation; countryCode: RU; stateProvince: Khanty-Mansiyskiy Avtonomnyy Okrug; county: Khanty-Mansiyskiy Rayon; locality: Mukhrino field station of YSU, 20 km SW from Khanty-Mansiysk; decimalLatitude: 60.891781; decimalLongitude: 68.684251; **Identification:** identifiedBy: Filippova, Nina|Zvyagina, Elena; dateIdentified: 2023-02-28; identificationRemarks: Identification based on morphological and molecular characters; **Event:** eventDate: 2022-08-19; habitat: Raised Sphagnum bog

#### Cortinarius collinitus

(Sowerby) Gray

9E2AA595-CA46-5818-B1E2-4C9B5C5FF805

##### Materials

- **Type status:** Other material. **Occurrence:** catalogNumber: YSU-F-05994; recordedBy: Filippova, Nina; associatedSequences: OQ366566; occurrenceID: B29EC05D-D24E-5CBF-BAD8-3D8143DEFD1C; **Location:** country: Russian Federation; countryCode: RU; stateProvince: Khanty-Mansiyskiy Avtonomnyy Okrug; county: Khanty-Mansiyskiy Rayon; locality: Mukhrino field station of YSU, 20 km SW from Khanty-Mansiysk; decimalLatitude: 60.892010; decimalLongitude: 68.682420; **Identification:** identifiedBy: Filippova, Nina|Zvyagina, Elena; dateIdentified: 2023-02-28; identificationRemarks: Identification based on morphological and molecular characters; **Event:** eventDate: 2015-08-15; habitat: Pine - dwarfshrubs - S. fuscum ombrotrophic bog
- **Type status:** Other material. **Occurrence:** catalogNumber: YSU-F-08467; recordedBy: Filippova, Nina; associatedSequences: OQ366567; occurrenceID: 3A02782C-5D1E-511C-9B52-CCE23AD7C5C1; **Location:** country: Russian Federation; countryCode: RU; stateProvince: Tomskaya Oblast'; county: Tomskiy Rayon; locality: Orlovka village vicinity, Chernoye lake; decimalLatitude: 56.878320; decimalLongitude: 84.665770; **Identification:** identifiedBy: Filippova, Nina|Zvyagina, Elena; dateIdentified: 2023-02-28; identificationRemarks: Identification based on morphological and molecular characters; **Event:** eventDate: 2018-08-22; habitat: Raised Pine-dwarfshrubs-Sphagnum bog
- **Type status:** Other material. **Occurrence:** catalogNumber: YSU-F-12173; recordedBy: Rudykina, Elena|Dobrynina, Alevtina; associatedSequences: OP866260; occurrenceID: 9688D5DC-7495-52EF-8997-815388848D18; **Location:** country: Russian Federation; countryCode: RU; stateProvince: Khanty-Mansiyskiy Avtonomnyy Okrug; county: Khanty-Mansiyskiy Rayon; locality: Mukhrino field station of YSU, 20 km SW from Khanty-Mansiysk; decimalLatitude: 60.891781; decimalLongitude: 68.684251; **Identification:** identifiedBy: Filippova, Nina|Zvyagina, Elena; dateIdentified: 2023-02-28; identificationRemarks: Identification based on morphological and molecular characters; **Event:** eventDate: 2022-08-19; habitat: Raised Sphagnum bog

#### Thaxterogaster comarostaphylidis

Ammirati, Halling & Garnica

A9C0632E-04EC-5225-B59C-4E0E5481C724

##### Materials

- **Type status:** Other material. **Occurrence:** catalogNumber: YSU-F-04388; recordedBy: Filippova, Nina; associatedSequences: OQ366583; occurrenceID: DE9BF282-30AE-5EC1-B782-40EC5A9C7CC6; **Location:** country: Russian Federation; countryCode: RU; stateProvince: Khanty-Mansiyskiy Avtonomnyy Okrug; county: Khanty-Mansiyskiy Rayon; locality: Mukhrino field station of YSU, 20 km SW from Khanty-Mansiysk; decimalLatitude: 60.892632; decimalLongitude: 68.677156; **Identification:** identifiedBy: Filippova, Nina|Zvyagina, Elena; dateIdentified: 2023-02-28; identificationRemarks: Identification based on morphological and molecular characters; **Event:** eventDate: 2013-08-23; habitat: Dwarfshrubs - sphagnum ombrotrophic bog
- **Type status:** Other material. **Occurrence:** catalogNumber: YSU-F-03949; recordedBy: Filippova, Nina; associatedSequences: OQ366584; occurrenceID: E908B388-6CDD-5497-8BD9-A8282E3990EC; **Location:** country: Russian Federation; countryCode: RU; stateProvince: Khanty-Mansiyskiy Avtonomnyy Okrug; county: Khanty-Mansiyskiy Rayon; locality: Mukhrino field station of YSU, 20 km SW from Khanty-Mansiysk; decimalLatitude: 60.889934; decimalLongitude: 68.700686; **Identification:** identifiedBy: Filippova, Nina|Zvyagina, Elena; dateIdentified: 2023-02-28; identificationRemarks: Identification based on morphological and molecular characters; **Event:** eventDate: 2012-09-01; habitat: Pine - dwarfshrubs - sphagnum bog (close to forest)

#### Cortinarius cruentiphyllus

Niskanen, Liimat., Kytöv., Ammirati, Dima, L. Albert & K.W.

8A4CBF69-9CB2-5476-9386-96AB9B315D81

##### Materials

- **Type status:** Other material. **Occurrence:** catalogNumber: YSU-F-12099; recordedBy: Filippova, Nina; associatedSequences: OP866240; occurrenceID: 623630B6-75D5-5D75-A3A8-AC4E54BCFEB1; **Location:** country: Russian Federation; countryCode: RU; stateProvince: Khanty-Mansiyskiy Avtonomnyy Okrug; county: Khanty-Mansiyskiy Rayon; locality: Shapsha village vicinity, 20 km E from Khanty-Mansiysk; decimalLatitude: 61.066410; decimalLongitude: 69.468030; **Identification:** identifiedBy: Filippova, Nina|Zvyagina, Elena; dateIdentified: 2023-02-28; identificationRemarks: Identification based on morphological and molecular characters; **Event:** eventDate: 2022-08-07; habitat: Raised Sphagnum bog

#### Cortinarius davemallochii

Ammirati, Niskanen & Liimat.

C4C55319-9319-5404-90FD-41E7C2538465

##### Materials

- **Type status:** Other material. **Occurrence:** catalogNumber: YSU-F-10097; recordedBy: Filippova, Nina; associatedSequences: OP866232; occurrenceID: CCEDB011-FEBC-5BB6-BB69-00CD6CCAD244; **Location:** country: Russian Federation; countryCode: RU; stateProvince: Khanty-Mansiyskiy Avtonomnyy Okrug; county: Khanty-Mansiyskiy Rayon; locality: Khanty-Mansiysk town vicinity; decimalLatitude: 60.891890; decimalLongitude: 68.682040; **Identification:** identifiedBy: Filippova, Nina|Zvyagina, Elena; dateIdentified: 2023-02-28; identificationRemarks: Identification based on morphological and molecular characters; **Event:** eventDate: 2020-07-28; habitat: Treed Pine-dwarfshrubs-Sphagnum bog
- **Type status:** Other material. **Occurrence:** catalogNumber: YSU-F-12122; recordedBy: Filippova, Nina|Rudykina, Elena; associatedSequences: OQ366568; occurrenceID: C2175434-49F0-5440-BF39-CCABA3B92DB7; **Location:** country: Russian Federation; countryCode: RU; stateProvince: Khanty-Mansiyskiy Avtonomnyy Okrug; county: Khanty-Mansiyskiy Rayon; locality: Mukhrino field station of YSU, 20 km SW from Khanty-Mansiysk; decimalLatitude: 60.891781; decimalLongitude: 68.684251; **Identification:** identifiedBy: Filippova, Nina|Zvyagina, Elena; dateIdentified: 2023-02-28; identificationRemarks: Identification based on morphological and molecular characters; **Event:** eventDate: 2022-08-04; habitat: Raised Sphagnum bog
- **Type status:** Other material. **Occurrence:** catalogNumber: YSU-F-12165; recordedBy: Rudykina, Elena|Dobrynina, Alevtina; associatedSequences: OP866253; occurrenceID: 3A50BEAC-C5F5-5084-837F-271F37B43B76; **Location:** country: Russian Federation; countryCode: RU; stateProvince: Khanty-Mansiyskiy Avtonomnyy Okrug; county: Khanty-Mansiyskiy Rayon; locality: Mukhrino field station of YSU, 20 km SW from Khanty-Mansiysk; decimalLatitude: 60.891781; decimalLongitude: 68.684251; **Identification:** identifiedBy: Filippova, Nina|Zvyagina, Elena; dateIdentified: 2023-02-28; identificationRemarks: Identification based on morphological and molecular characters; **Event:** eventDate: 2022-08-19; habitat: Raised Sphagnum bog

#### Cortinarius glandicolor

(Fr.) Fr.

A2190288-0B81-526F-AF77-B857F70F5740

##### Materials

- **Type status:** Other material. **Occurrence:** catalogNumber: YSU-F-05991; recordedBy: Filippova, Nina; associatedSequences: OP866209; occurrenceID: 868F99D4-38E6-55A7-A3AA-FBD113CCDF9C; **Location:** country: Russian Federation; countryCode: RU; stateProvince: Khanty-Mansiyskiy Avtonomnyy Okrug; county: Khanty-Mansiyskiy Rayon; locality: Mukhrino field station of YSU, 20 km SW from Khanty-Mansiysk; decimalLatitude: 60.892010; decimalLongitude: 68.682420; **Identification:** identifiedBy: Filippova, Nina|Zvyagina, Elena; dateIdentified: 2023-02-28; identificationRemarks: Identification based on morphological and molecular characters; **Event:** eventDate: 2015-08-15; habitat: Pine - dwarfshrubs - S. fuscum ombrotrophic bog

#### Cortinarius kauffmanianus

A.H.Sm.

E5FCDC8A-50E1-5B8C-9D01-69A7C9652E8B

##### Materials

- **Type status:** Other material. **Occurrence:** catalogNumber: YSU-F-06276; recordedBy: Filippova, Nina; associatedSequences: OQ366586; occurrenceID: 18858B58-E0C4-5D1A-BE1C-38D87475D1D8; **Location:** country: Russian Federation; countryCode: RU; stateProvince: Khanty-Mansiyskiy Avtonomnyy Okrug; county: Khanty-Mansiyskiy Rayon; locality: Mukhrino field station of YSU, 20 km SW from Khanty-Mansiysk; decimalLatitude: 60.892010; decimalLongitude: 68.682420; **Identification:** identifiedBy: Filippova, Nina|Zvyagina, Elena; dateIdentified: 2023-02-28; identificationRemarks: Identification based on morphological and molecular characters; **Event:** eventDate: 2015-08-24; habitat: Pine - dwarfshrubs - S. fuscum ombrotrophic bog
- **Type status:** Other material. **Occurrence:** catalogNumber: YSU-F-07313; recordedBy: Filippova, Nina; associatedSequences: OP866216; occurrenceID: 78A4F1C9-21C4-556C-A603-1536CA231141; **Location:** country: Russian Federation; countryCode: RU; stateProvince: Khanty-Mansiyskiy Avtonomnyy Okrug; county: Khanty-Mansiyskiy Rayon; locality: Mukhrino field station of YSU, 20 km SW from Khanty-Mansiysk; decimalLatitude: 60.891781; decimalLongitude: 68.684251; **Identification:** identifiedBy: Filippova, Nina|Zvyagina, Elena; dateIdentified: 2023-02-28; identificationRemarks: Identification based on morphological and molecular characters; **Event:** eventDate: 2016-09-05; habitat: Pine-dwarfshrubs-S.fuscum ombrotrophic bog
- **Type status:** Other material. **Occurrence:** catalogNumber: YSU-F-07314; recordedBy: Filippova, Nina; associatedSequences: OQ366587; occurrenceID: FD1B18D1-0615-5DB0-B753-225C39AAD587; **Location:** country: Russian Federation; countryCode: RU; stateProvince: Khanty-Mansiyskiy Avtonomnyy Okrug; county: Khanty-Mansiyskiy Rayon; locality: Mukhrino field station of YSU, 20 km SW from Khanty-Mansiysk; decimalLatitude: 60.891781; decimalLongitude: 68.684251; **Identification:** identifiedBy: Filippova, Nina|Zvyagina, Elena; dateIdentified: 2023-02-28; identificationRemarks: Identification based on morphological and molecular characters; **Event:** eventDate: 2016-09-05; habitat: Pine-dwarfshrubs-S.fuscum ombrotrophic bog
- **Type status:** Other material. **Occurrence:** catalogNumber: YSU-F-12725; recordedBy: Filippova, Nina; associatedSequences: OQ366585; occurrenceID: 633B97EA-9FE9-58C5-A6BE-93C1CB2C6BBC; **Location:** country: Russian Federation; countryCode: RU; stateProvince: Tomskaya Oblast'; county: Tomskiy Rayon; locality: Orlovka village vicinity, Chernoye lake; decimalLatitude: 56.878320; decimalLongitude: 84.665770; **Identification:** identifiedBy: Filippova, Nina|Zvyagina, Elena; dateIdentified: 2023-02-28; identificationRemarks: Identification based on morphological and molecular characters; **Event:** eventDate: 2018-08-22; habitat: Raised Pine-dwarfshrubs-Sphagnum bog
- **Type status:** Other material. **Occurrence:** catalogNumber: YSU-F-08628; recordedBy: Filippova, Nina; associatedSequences: OP866229; occurrenceID: E374A934-EA57-5C90-904B-F227281A589C; **Location:** country: Russian Federation; countryCode: RU; stateProvince: Khanty-Mansiyskiy Avtonomnyy Okrug; county: Khanty-Mansiyskiy Rayon; locality: Mukhrino field station of YSU, 20 km SW from Khanty-Mansiysk; decimalLatitude: 60.891781; decimalLongitude: 68.684251; **Identification:** identifiedBy: Filippova, Nina|Zvyagina, Elena; dateIdentified: 2023-02-28; identificationRemarks: Identification based on morphological and molecular characters; **Event:** eventDate: 2018-09-04; habitat: Raised Sphagnum bog
- **Type status:** Other material. **Occurrence:** catalogNumber: YSU-F-08358; recordedBy: Filippova, Nina; associatedSequences: OP866225; occurrenceID: DBE77E8E-0A93-5611-A340-4D69AEB0C82A; **Location:** country: Russian Federation; countryCode: RU; stateProvince: Khanty-Mansiyskiy Avtonomnyy Okrug; county: Khanty-Mansiyskiy Rayon; locality: Mukhrino field station of YSU, 20 km SW from Khanty-Mansiysk; decimalLatitude: 60.891781; decimalLongitude: 68.684251; **Identification:** identifiedBy: Filippova, Nina|Zvyagina, Elena; dateIdentified: 2023-02-28; identificationRemarks: Identification based on morphological and molecular characters; **Event:** eventDate: 2018-08-28; habitat: Treed Pine-dwarfshrubs-Sphagnum bog
- **Type status:** Other material. **Occurrence:** catalogNumber: YSU-F-12170; recordedBy: Rudykina, Elena|Dobrynina, Alevtina; associatedSequences: OP866258; occurrenceID: 23739D82-E509-5BD7-9FF8-72C85C8779B9; **Location:** country: Russian Federation; countryCode: RU; stateProvince: Khanty-Mansiyskiy Avtonomnyy Okrug; county: Khanty-Mansiyskiy Rayon; locality: Mukhrino field station of YSU, 20 km SW from Khanty-Mansiysk; decimalLatitude: 60.891781; decimalLongitude: 68.684251; **Identification:** identifiedBy: Filippova, Nina|Zvyagina, Elena; dateIdentified: 2023-02-28; identificationRemarks: Identification based on morphological and molecular characters; **Event:** eventDate: 2022-08-19; habitat: Raised Sphagnum bog
- **Type status:** Other material. **Occurrence:** recordedBy: Filippova, Nina; associatedSequences: OQ396710; occurrenceID: FBF4CF3B-1622-59EB-8837-264005AA60EA; **Location:** country: Russian Federation; countryCode: RU; stateProvince: Khanty-Mansiyskiy Avtonomnyy Okrug; county: Khanty-Mansiyskiy Rayon; decimalLatitude: 60.891780; decimalLongitude: 68.684250; **Identification:** identifiedBy: Filippova, Nina|Zvyagina, Elena; dateIdentified: 2023-02-28; identificationRemarks: Identification based on morphological and molecular characters; **Event:** eventDate: 2022-08-31; habitat: Raised Sphagnum bog
- **Type status:** Other material. **Occurrence:** catalogNumber: YSU-F-12131; recordedBy: Filippova, Nina|Rudykina, Elena; associatedSequences: OP866250; occurrenceID: 54BCFD0E-650F-5845-AFB3-9839F0AE3BFE; **Location:** country: Russian Federation; countryCode: RU; stateProvince: Khanty-Mansiyskiy Avtonomnyy Okrug; county: Khanty-Mansiyskiy Rayon; locality: Mukhrino field station of YSU, 20 km SW from Khanty-Mansiysk; decimalLatitude: 60.891781; decimalLongitude: 68.684251; **Identification:** identifiedBy: Filippova, Nina|Zvyagina, Elena; dateIdentified: 2023-02-28; identificationRemarks: Identification based on morphological and molecular characters; **Event:** eventDate: 2022-08-04; habitat: Raised Sphagnum bog

#### Cortinarius lindstroemii

Niskanen, Kytov. & Liimat.

F413E362-006C-559D-BAD7-ACFEC2CE8D0B

##### Materials

- **Type status:** Other material. **Occurrence:** catalogNumber: YSU-F-12167; recordedBy: Rudykina, Elena|Dobrynina, Alevtina; associatedSequences: OP866255; occurrenceID: 481657C2-5379-5456-9FC4-FCA3F346F010; **Location:** country: Russian Federation; countryCode: RU; stateProvince: Khanty-Mansiyskiy Avtonomnyy Okrug; county: Khanty-Mansiyskiy Rayon; locality: Mukhrino field station of YSU, 20 km SW from Khanty-Mansiysk; decimalLatitude: 60.891781; decimalLongitude: 68.684251; **Identification:** identifiedBy: Filippova, Nina|Zvyagina, Elena; dateIdentified: 2023-02-28; identificationRemarks: Identification based on morphological and molecular characters; **Event:** eventDate: 2022-08-19; habitat: Raised Sphagnum bog

#### Cortinarius malachius

(Fr.) Fr.

C08686E8-513F-5B80-A665-BD5773D60FFC

##### Materials

- **Type status:** Other material. **Occurrence:** catalogNumber: YSU-F-06275; recordedBy: Filippova, Nina; associatedSequences: OQ366597; occurrenceID: 068F9024-F3ED-54EA-B940-0C1A66C35911; **Location:** country: Russian Federation; countryCode: RU; stateProvince: Khanty-Mansiyskiy Avtonomnyy Okrug; county: Khanty-Mansiyskiy Rayon; locality: Mukhrino field station of YSU, 20 km SW from Khanty-Mansiysk; decimalLatitude: 60.892010; decimalLongitude: 68.682420; **Identification:** identifiedBy: Filippova, Nina|Zvyagina, Elena; dateIdentified: 2023-02-28; identificationRemarks: Identification based on morphological and molecular characters; **Event:** eventDate: 2015-08-24; habitat: Pine - dwarfshrubs - S. fuscum ombrotrophic bog
- **Type status:** Other material. **Occurrence:** catalogNumber: YSU-F-03938; recordedBy: Filippova, Nina; associatedSequences: OQ366598; occurrenceID: 00633617-C1F9-523D-BBA1-380B51D1337D; **Location:** country: Russian Federation; countryCode: RU; stateProvince: Khanty-Mansiyskiy Avtonomnyy Okrug; county: Khanty-Mansiyskiy Rayon; locality: Mukhrino field station of YSU, 20 km SW from Khanty-Mansiysk; decimalLatitude: 60.889934; decimalLongitude: 68.700686; **Identification:** identifiedBy: Filippova, Nina|Zvyagina, Elena; dateIdentified: 2023-02-28; identificationRemarks: Identification based on morphological and molecular characters; **Event:** eventDate: 2012-09-01; habitat: Pine - dwarfshrubs - sphagnum bog (close to forest)

#### Thaxterogaster pinophilus

Soop

16889A91-179C-55CF-967F-480AF432C9F0

##### Materials

- **Type status:** Other material. **Occurrence:** catalogNumber: YSU-F-07312; recordedBy: Filippova, Nina; associatedSequences: OQ366578; occurrenceID: 62B63D0B-CB2E-57E4-82B9-D6F2B0666695; **Location:** country: Russian Federation; countryCode: RU; stateProvince: Khanty-Mansiyskiy Avtonomnyy Okrug; county: Khanty-Mansiyskiy Rayon; locality: Mukhrino field station of YSU, 20 km SW from Khanty-Mansiysk; decimalLatitude: 60.891781; decimalLongitude: 68.684251; **Identification:** identifiedBy: Filippova, Nina|Zvyagina, Elena; dateIdentified: 2023-02-28; identificationRemarks: Identification based on morphological and molecular characters; **Event:** eventDate: 2016-09-05; habitat: Pine-dwarfshrubs-S.fuscum ombrotrophic bog

#### Cortinarius quarciticus

H.Lindstr.

3290010A-6BA3-52F0-A177-2FA0F8ED489C

##### Materials

- **Type status:** Other material. **Occurrence:** catalogNumber: YSU-F-08477; recordedBy: Filippova, Nina; associatedSequences: OQ366595; occurrenceID: A8CDBF38-1D00-5DF2-9490-10C77491FABD; **Location:** country: Russian Federation; countryCode: RU; stateProvince: Tomskaya Oblast'; county: Tomskiy Rayon; locality: Orlovka village vicinity, Chernoye lake; decimalLatitude: 56.878320; decimalLongitude: 84.665770; **Identification:** identifiedBy: Filippova, Nina|Zvyagina, Elena; dateIdentified: 2023-02-28; identificationRemarks: Identification based on morphological and molecular characters; **Event:** eventDate: 2018-08-22; habitat: Raised Pine-dwarfshrubs-Sphagnum bog
- **Type status:** Other material. **Occurrence:** catalogNumber: YSU-F-10532; recordedBy: Filippova, Nina; associatedSequences: OQ366594; occurrenceID: B4D043FF-69D2-5DEA-9996-5BFDE77ED749; **Location:** country: Russian Federation; countryCode: RU; stateProvince: Khanty-Mansiyskiy Avtonomnyy Okrug; county: Khanty-Mansiyskiy Rayon; locality: Khanty-Mansiysk town vicinity; decimalLatitude: 60.891900; decimalLongitude: 68.682260; **Identification:** identifiedBy: Filippova, Nina|Zvyagina, Elena; dateIdentified: 2023-02-28; identificationRemarks: Identification based on morphological and molecular characters; **Event:** eventDate: 2020-09-08; habitat: Raised Sphagnum bog
- **Type status:** Other material. **Occurrence:** catalogNumber: YSU-F-08353; recordedBy: Filippova, Nina; associatedSequences: OQ366593; occurrenceID: 88D328D5-44D5-554A-9EDC-EB24C19FFA0C; **Location:** country: Russian Federation; countryCode: RU; stateProvince: Khanty-Mansiyskiy Avtonomnyy Okrug; county: Khanty-Mansiyskiy Rayon; locality: Mukhrino field station of YSU, 20 km SW from Khanty-Mansiysk; decimalLatitude: 60.891781; decimalLongitude: 68.684251; **Identification:** identifiedBy: Filippova, Nina|Zvyagina, Elena; dateIdentified: 2023-02-28; identificationRemarks: Identification based on morphological and molecular characters; **Event:** eventDate: 2018-08-28; habitat: Treed Pine-dwarfshrubs-Sphagnum bog

#### Cortinarius rubellus

Cooke

9C850A4B-E6ED-5C0F-A26B-544EA1174640

##### Materials

- **Type status:** Other material. **Occurrence:** catalogNumber: YSU-F-05844; recordedBy: Filippova, Nina; associatedSequences: OQ366581; occurrenceID: C9DC8601-0FD9-5EAF-A7EA-D829434C43AE; **Location:** country: Russian Federation; countryCode: RU; stateProvince: Khanty-Mansiyskiy Avtonomnyy Okrug; county: Khanty-Mansiyskiy Rayon; locality: Mukhrino field station of YSU, 20 km SW from Khanty-Mansiysk; decimalLatitude: 60.891781; decimalLongitude: 68.684251; **Identification:** identifiedBy: Filippova, Nina|Zvyagina, Elena; dateIdentified: 2023-02-28; identificationRemarks: Identification based on morphological and molecular characters; **Event:** eventDate: 2015-08-08; habitat: Pine - dwarfshrubs - S. fuscum ombrotrophic bog
- **Type status:** Other material. **Occurrence:** catalogNumber: YSU-F-07913; recordedBy: Filippova, Nina; associatedSequences: OQ366582; occurrenceID: FD9EA0EF-A07C-5CCA-B1C4-166EF12501C7; **Location:** country: Russian Federation; countryCode: RU; stateProvince: Khanty-Mansiyskiy Avtonomnyy Okrug; county: Khanty-Mansiyskiy Rayon; locality: Mukhrino field station of YSU, 20 km SW from Khanty-Mansiysk; decimalLatitude: 60.891781; decimalLongitude: 68.684251; **Identification:** identifiedBy: Filippova, Nina|Zvyagina, Elena; dateIdentified: 2023-02-28; identificationRemarks: Identification based on morphological and molecular characters; **Event:** eventDate: 2017-08-07; habitat: Pine-dwarfshrubs-S.fuscum ombrotrophic bog

#### Cortinarius scaurus

(Fr.) Fr.

7047D9E1-4DC7-5CFA-8614-22BDCC278476

##### Materials

- **Type status:** Other material. **Occurrence:** catalogNumber: YSU-F-03821; recordedBy: Filippova, Nina; associatedSequences: OQ366576; occurrenceID: 4BE00B63-C6E2-5298-A268-E6F4C13F53BA; **Location:** country: Russian Federation; countryCode: RU; stateProvince: Khanty-Mansiyskiy Avtonomnyy Okrug; county: Khanty-Mansiyskiy Rayon; locality: Chistoe bog, 20 km E from Khanty-Mansiysk; decimalLatitude: 61.046340; decimalLongitude: 69.440660; **Identification:** identifiedBy: Filippova, Nina|Zvyagina, Elena; dateIdentified: 2023-02-28; identificationRemarks: Identification based on morphological and molecular characters; **Event:** eventDate: 2012-08-22; habitat: Pine - dwarfshrubs - sphagnum ombrotrophic bog
- **Type status:** Other material. **Occurrence:** catalogNumber: YSU-F-08356; recordedBy: Filippova, Nina; associatedSequences: OQ366577; occurrenceID: 8E225DAD-017D-512B-8374-393C461DD2A9; **Location:** country: Russian Federation; countryCode: RU; stateProvince: Khanty-Mansiyskiy Avtonomnyy Okrug; county: Khanty-Mansiyskiy Rayon; locality: Mukhrino field station of YSU, 20 km SW from Khanty-Mansiysk; decimalLatitude: 60.891781; decimalLongitude: 68.684251; **Identification:** identifiedBy: Filippova, Nina|Zvyagina, Elena; dateIdentified: 2023-02-28; identificationRemarks: Identification based on morphological and molecular characters; **Event:** eventDate: 2018-08-28; habitat: Treed Pine-dwarfshrubs-Sphagnum bog

#### Cortinarius semisanguineus

(Fr.) Gillet

297817C4-59E0-58C3-B36E-84FAF1E79760

##### Materials

- **Type status:** Other material. **Occurrence:** catalogNumber: YSU-F-04089; recordedBy: Filippova, Nina; associatedSequences: OQ366571; occurrenceID: 4868C501-8E37-50A8-AB59-3009F2BCEB38; **Location:** country: Russian Federation; countryCode: RU; stateProvince: Khanty-Mansiyskiy Avtonomnyy Okrug; county: Khanty-Mansiyskiy Rayon; locality: Mukhrino field station of YSU, 20 km SW from Khanty-Mansiysk; decimalLatitude: 60.892022; decimalLongitude: 68.691502; **Identification:** identifiedBy: Filippova, Nina|Zvyagina, Elena; dateIdentified: 2023-02-28; identificationRemarks: Identification based on morphological and molecular characters; **Event:** eventDate: 2012-09-09; habitat: Pine - dwarfshrubs - sphagnum ombrotrophic bog
- **Type status:** Other material. **Occurrence:** catalogNumber: YSU-F-10539; recordedBy: Filippova, Nina; associatedSequences: OQ366570; occurrenceID: B4C5062C-75F0-55A4-8523-05487A24BE4B; **Location:** country: Russian Federation; countryCode: RU; stateProvince: Khanty-Mansiyskiy Avtonomnyy Okrug; county: Khanty-Mansiyskiy Rayon; locality: Khanty-Mansiysk town vicinity; decimalLatitude: 60.891900; decimalLongitude: 68.682260; **Identification:** identifiedBy: Filippova, Nina|Zvyagina, Elena; dateIdentified: 2023-02-28; identificationRemarks: Identification based on morphological and molecular characters; **Event:** eventDate: 2020-09-08; habitat: Raised Sphagnum bog

#### Cortinarius sphagnoravus

Liimat., Kytöv., Niskanen & Ammirati

F8C9E686-CACD-5C55-A17D-2A975EAF30D9

##### Materials

- **Type status:** Other material. **Occurrence:** catalogNumber: YSU-F-04378; recordedBy: Filippova, Nina; associatedSequences: OQ366590; occurrenceID: 2DB3B76D-2330-5650-A98C-DDA155DC2B89; **Location:** country: Russian Federation; countryCode: RU; stateProvince: Khanty-Mansiyskiy Avtonomnyy Okrug; county: Khanty-Mansiyskiy Rayon; locality: Mukhrino field station of YSU, 20 km SW from Khanty-Mansiysk; decimalLatitude: 60.892632; decimalLongitude: 68.677156; **Identification:** identifiedBy: Filippova, Nina|Zvyagina, Elena; dateIdentified: 2023-02-28; identificationRemarks: Identification based on morphological and molecular characters; **Event:** eventDate: 2013-08-22; habitat: Dwarfshrubs - sphagnum ombrotrophic bog
- **Type status:** Other material. **Occurrence:** catalogNumber: YSU-F-06282; recordedBy: Filippova, Nina; associatedSequences: OP866213; occurrenceID: 414EC551-167F-5168-B7EA-163F5D8C5008; **Location:** country: Russian Federation; countryCode: RU; stateProvince: Khanty-Mansiyskiy Avtonomnyy Okrug; county: Khanty-Mansiyskiy Rayon; locality: Mukhrino field station of YSU, 20 km SW from Khanty-Mansiysk; decimalLatitude: 60.892010; decimalLongitude: 68.682420; **Identification:** identifiedBy: Filippova, Nina|Zvyagina, Elena; dateIdentified: 2023-02-28; identificationRemarks: Identification based on morphological and molecular characters; **Event:** eventDate: 2015-08-24; habitat: Pine - dwarfshrubs - S. fuscum ombrotrophic bog
- **Type status:** Other material. **Occurrence:** catalogNumber: YSU-F-05840; recordedBy: Filippova, Nina; associatedSequences: OP866207; occurrenceID: 58F6D14A-A81A-5CF8-9B5C-C1DCB6764A6C; **Location:** country: Russian Federation; countryCode: RU; stateProvince: Khanty-Mansiyskiy Avtonomnyy Okrug; county: Khanty-Mansiyskiy Rayon; locality: Mukhrino field station of YSU, 20 km SW from Khanty-Mansiysk; decimalLatitude: 60.891781; decimalLongitude: 68.684251; **Identification:** identifiedBy: Filippova, Nina|Zvyagina, Elena; dateIdentified: 2023-02-28; identificationRemarks: Identification based on morphological and molecular characters; **Event:** eventDate: 2015-08-08; habitat: Pine - dwarfshrubs - S. fuscum ombrotrophic bog
- **Type status:** Other material. **Occurrence:** catalogNumber: YSU-F-03985; recordedBy: Filippova, Nina; associatedSequences: OP866187; occurrenceID: 7CDCB481-E72D-5FE3-BEEA-73565EA1E411; **Location:** country: Russian Federation; countryCode: RU; stateProvince: Khanty-Mansiyskiy Avtonomnyy Okrug; county: Khanty-Mansiyskiy Rayon; locality: Mukhrino field station of YSU, 20 km SW from Khanty-Mansiysk; decimalLatitude: 60.889934; decimalLongitude: 68.700686; **Identification:** identifiedBy: Filippova, Nina|Zvyagina, Elena; dateIdentified: 2023-02-28; identificationRemarks: Identification based on morphological and molecular characters; **Event:** eventDate: 2012-09-02; habitat: Pine - dwarfshrubs - sphagnum bog (close to forest)
- **Type status:** Other material. **Occurrence:** catalogNumber: YSU-F-08472; recordedBy: Filippova, Nina; associatedSequences: OP866227; occurrenceID: 65166951-E7A2-54AD-8B09-FAC2A611395D; **Location:** country: Russian Federation; countryCode: RU; stateProvince: Tomskaya Oblast'; county: Tomskiy Rayon; locality: Orlovka village vicinity, Chernoye lake; decimalLatitude: 56.878320; decimalLongitude: 84.665770; **Identification:** identifiedBy: Filippova, Nina|Zvyagina, Elena; dateIdentified: 2023-02-28; identificationRemarks: Identification based on morphological and molecular characters; **Event:** eventDate: 2018-08-22; habitat: Raised Pine-dwarfshrubs-Sphagnum bog
- **Type status:** Other material. **Occurrence:** catalogNumber: YSU-F-10537; recordedBy: Filippova, Nina; associatedSequences: OP866237; occurrenceID: F6354D21-8173-55F6-ACF9-EAC329B62E80; **Location:** country: Russian Federation; countryCode: RU; stateProvince: Khanty-Mansiyskiy Avtonomnyy Okrug; county: Khanty-Mansiyskiy Rayon; locality: Khanty-Mansiysk town vicinity; decimalLatitude: 60.891900; decimalLongitude: 68.682260; **Identification:** identifiedBy: Filippova, Nina|Zvyagina, Elena; dateIdentified: 2023-02-28; identificationRemarks: Identification based on morphological and molecular characters; **Event:** eventDate: 2020-09-08; habitat: Raised Sphagnum bog
- **Type status:** Other material. **Occurrence:** catalogNumber: YSU-F-12169; recordedBy: Rudykina, Elena|Dobrynina, Alevtina; associatedSequences: OP866257; occurrenceID: CA7BCABB-D804-560B-8497-039C82AAF095; **Location:** country: Russian Federation; countryCode: RU; stateProvince: Khanty-Mansiyskiy Avtonomnyy Okrug; county: Khanty-Mansiyskiy Rayon; locality: Mukhrino field station of YSU, 20 km SW from Khanty-Mansiysk; decimalLatitude: 60.891781; decimalLongitude: 68.684251; **Identification:** identifiedBy: Filippova, Nina|Zvyagina, Elena; dateIdentified: 2023-02-28; identificationRemarks: Identification based on morphological and molecular characters; **Event:** eventDate: 2022-08-19; habitat: Raised Sphagnum bog
- **Type status:** Other material. **Occurrence:** catalogNumber: YSU-F-12172; recordedBy: Rudykina, Elena|Dobrynina, Alevtina; associatedSequences: OP866259; occurrenceID: B921CABE-5EA1-55BA-B990-415FAA7D0735; **Location:** country: Russian Federation; countryCode: RU; stateProvince: Khanty-Mansiyskiy Avtonomnyy Okrug; county: Khanty-Mansiyskiy Rayon; locality: Mukhrino field station of YSU, 20 km SW from Khanty-Mansiysk; decimalLatitude: 60.891781; decimalLongitude: 68.684251; **Identification:** identifiedBy: Filippova, Nina|Zvyagina, Elena; dateIdentified: 2023-02-28; identificationRemarks: Identification based on morphological and molecular characters; **Event:** eventDate: 2022-08-19; habitat: Raised Sphagnum bog

#### Cortinarius suberi

Soop

90FF1B09-D829-5FDE-94C4-3485B80DE8E6

##### Materials

- **Type status:** Other material. **Occurrence:** catalogNumber: YSU-F-04407; recordedBy: Filippova, Nina; associatedSequences: OQ366592; occurrenceID: 08CBBB5A-B56D-531F-9DC2-0945F9DB5E80; **Location:** country: Russian Federation; countryCode: RU; stateProvince: Khanty-Mansiyskiy Avtonomnyy Okrug; county: Khanty-Mansiyskiy Rayon; locality: Mukhrino field station of YSU, 20 km SW from Khanty-Mansiysk; decimalLatitude: 60.892632; decimalLongitude: 68.677156; **Identification:** identifiedBy: Filippova, Nina|Zvyagina, Elena; dateIdentified: 2023-02-28; identificationRemarks: Identification based on morphological and molecular characters; **Event:** eventDate: 2013-09-02; habitat: Dwarfshrubs - sphagnum ombrotrophic bog

#### Cortinarius tenuifulvescens

Kytöv., Niskanen & Liimat.

31D45FDD-5447-5BD6-A876-343FD6AB1A9D

##### Materials

- **Type status:** Other material. **Occurrence:** catalogNumber: YSU-F-06281; recordedBy: Filippova, Nina; associatedSequences: OP866212; occurrenceID: BF9D0746-D25C-5276-9DEA-D38E6D1977E6; **Location:** country: Russian Federation; countryCode: RU; stateProvince: Khanty-Mansiyskiy Avtonomnyy Okrug; county: Khanty-Mansiyskiy Rayon; locality: Mukhrino field station of YSU, 20 km SW from Khanty-Mansiysk; decimalLatitude: 60.892010; decimalLongitude: 68.682420; **Identification:** identifiedBy: Filippova, Nina|Zvyagina, Elena; dateIdentified: 2023-02-28; identificationRemarks: Identification based on morphological and molecular characters; **Event:** eventDate: 2015-08-24; habitat: Pine - dwarfshrubs - S. fuscum ombrotrophic bog
- **Type status:** Other material. **Occurrence:** catalogNumber: YSU-F-04092; recordedBy: Filippova, Nina; associatedSequences: OQ366569; occurrenceID: 4DACD1A3-5B5A-5A71-AEE0-4394F10348D2; **Location:** country: Russian Federation; countryCode: RU; stateProvince: Khanty-Mansiyskiy Avtonomnyy Okrug; county: Khanty-Mansiyskiy Rayon; locality: Mukhrino field station of YSU, 20 km SW from Khanty-Mansiysk; decimalLatitude: 60.892022; decimalLongitude: 68.691502; **Identification:** identifiedBy: Filippova, Nina|Zvyagina, Elena; dateIdentified: 2023-02-28; identificationRemarks: Identification based on morphological and molecular characters; **Event:** eventDate: 2012-09-09; habitat: Pine - dwarfshrubs - sphagnum ombrotrophic bog

#### Cortinarius biformis

Fr.

35FED5AC-3C33-5723-B353-7F5371AAAD03

##### Materials

- **Type status:** Other material. **Occurrence:** catalogNumber: YSU-F-07592; recordedBy: Filippova, Nina; associatedSequences: OQ366596; occurrenceID: 1430F8F0-3D01-56E3-A3B7-7FD82C873ED8; **Location:** country: Russian Federation; countryCode: RU; stateProvince: Khanty-Mansiyskiy Avtonomnyy Okrug; county: Khanty-Mansiyskiy Rayon; locality: Mukhrino field station of YSU, 20 km SW from Khanty-Mansiysk; decimalLatitude: 60.891781; decimalLongitude: 68.684251; **Identification:** identifiedBy: Filippova, Nina|Zvyagina, Elena; dateIdentified: 2023-02-28; identificationRemarks: Identification based on morphological and molecular characters; **Event:** eventDate: 2016-09-20; habitat: Pine-dwarfshrubs-S.fuscum ombrotrophic bog

#### Cortinarius venustus

P.Karst.

0B59F433-8481-502E-ACAB-D6894666D7AF

##### Materials

- **Type status:** Other material. **Occurrence:** catalogNumber: YSU-F-07591; recordedBy: Filippova, Nina; associatedSequences: OQ366588; occurrenceID: 9EE21C14-1725-5D3A-9250-F82642DCB58A; **Location:** country: Russian Federation; countryCode: RU; stateProvince: Khanty-Mansiyskiy Avtonomnyy Okrug; county: Khanty-Mansiyskiy Rayon; locality: Mukhrino field station of YSU, 20 km SW from Khanty-Mansiysk; decimalLatitude: 60.891781; decimalLongitude: 68.684251; **Identification:** identifiedBy: Filippova, Nina|Zvyagina, Elena; dateIdentified: 2023-02-28; identificationRemarks: Identification based on morphological and molecular characters; **Event:** eventDate: 2016-09-20; habitat: Pine-dwarfshrubs-S.fuscum ombrotrophic bog

#### Cortinarius sp.

8C8329A0-5695-5500-93A1-169D54E1E24A

##### Materials

- **Type status:** Other material. **Occurrence:** catalogNumber: YSU-F-04376; recordedBy: Filippova, Nina; associatedSequences: OQ406266; occurrenceID: 7C47453D-9D2D-5575-83BE-137598B1BF9D; **Location:** country: Russian Federation; countryCode: RU; stateProvince: Khanty-Mansiyskiy Avtonomnyy Okrug; county: Khanty-Mansiyskiy Rayon; locality: Mukhrino field station of YSU, 20 km SW from Khanty-Mansiysk; decimalLatitude: 60.892632; decimalLongitude: 68.677156; **Identification:** identifiedBy: Filippova, Nina|Zvyagina, Elena; dateIdentified: 2023-02-28; identificationRemarks: Identification based on morphological and molecular characters; **Event:** eventDate: 2013-08-22; habitat: Dwarfshrubs - sphagnum ombrotrophic bog

#### Cortinarius sp. 1

9F0CC674-74DD-597A-9A28-B14F328C7175

##### Materials

- **Type status:** Other material. **Occurrence:** catalogNumber: YSU-F-07112; recordedBy: Filippova, Nina; associatedSequences: OQ366573; occurrenceID: 68878D77-BC55-5D57-A666-354EBD4A5928; **Location:** country: Russian Federation; countryCode: RU; stateProvince: Khanty-Mansiyskiy Avtonomnyy Okrug; county: Khanty-Mansiyskiy Rayon; locality: Mukhrino field station of YSU, 20 km SW from Khanty-Mansiysk; decimalLatitude: 60.891781; decimalLongitude: 68.684251; **Identification:** identifiedBy: Filippova, Nina|Zvyagina, Elena; dateIdentified: 2023-02-28; identificationRemarks: Identification based on morphological and molecular characters; **Event:** eventDate: 2016-08-13; habitat: Pine-dwarfshrubs-S.fuscum ombrotrophic bog
- **Type status:** Other material. **Occurrence:** catalogNumber: YSU-F-12171; recordedBy: Rudykina, Elena|Dobrynina, Alevtina; associatedSequences: OQ366572; occurrenceID: B56EB31D-5D54-572D-A47B-2D55DAF9C20F; **Location:** country: Russian Federation; countryCode: RU; stateProvince: Khanty-Mansiyskiy Avtonomnyy Okrug; county: Khanty-Mansiyskiy Rayon; locality: Mukhrino field station of YSU, 20 km SW from Khanty-Mansiysk; decimalLatitude: 60.891781; decimalLongitude: 68.684251; **Identification:** identifiedBy: Filippova, Nina|Zvyagina, Elena; dateIdentified: 2023-02-28; identificationRemarks: Identification based on morphological and molecular characters; **Event:** eventDate: 2022-08-19; habitat: Raised Sphagnum bog

#### Cortinarius sp. 2

2DAC4B16-974B-50C3-8661-041D5D72E08A

##### Materials

- **Type status:** Other material. **Occurrence:** catalogNumber: YSU-F-06488; recordedBy: Filippova, Nina; associatedSequences: OQ366574; occurrenceID: 2078BD8B-0F28-5DDC-B4CC-B121888CFBAF; **Location:** country: Russian Federation; countryCode: RU; stateProvince: Khanty-Mansiyskiy Avtonomnyy Okrug; county: Khanty-Mansiyskiy Rayon; locality: Mukhrino field station of YSU, 20 km SW from Khanty-Mansiysk; decimalLatitude: 60.892010; decimalLongitude: 68.682420; **Identification:** identifiedBy: Filippova, Nina|Zvyagina, Elena; dateIdentified: 2023-02-28; identificationRemarks: Identification based on morphological and molecular characters; **Event:** eventDate: 2015-09-05; habitat: Pine - dwarfshrubs - S. fuscum ombrotrophic bog

#### Cortinarius sp. 3

DFF7D6C3-E46E-5EE0-BA5C-E4305FC0A2A8

##### Materials

- **Type status:** Other material. **Occurrence:** catalogNumber: YSU-F-05845; recordedBy: Filippova, Nina; associatedSequences: OQ366580; occurrenceID: EEA2E476-4939-5F99-B356-0A1CC75B8147; **Location:** country: Russian Federation; countryCode: RU; stateProvince: Khanty-Mansiyskiy Avtonomnyy Okrug; county: Khanty-Mansiyskiy Rayon; locality: Mukhrino field station of YSU, 20 km SW from Khanty-Mansiysk; decimalLatitude: 60.891781; decimalLongitude: 68.684251; **Identification:** identifiedBy: Filippova, Nina|Zvyagina, Elena; dateIdentified: 2023-02-28; identificationRemarks: Identification based on morphological and molecular characters; **Event:** eventDate: 2015-08-08; habitat: Pine - dwarfshrubs - S. fuscum ombrotrophic bog

#### Cortinarius sp. 4

BCF01F2C-976E-5DC1-8F61-6CB5E496679A

##### Materials

- **Type status:** Other material. **Occurrence:** catalogNumber: YSU-F-04424; recordedBy: Filippova, Nina; associatedSequences: OQ366591; occurrenceID: 7E0DBF71-3B71-5CCE-893E-06B51D590449; **Location:** country: Russian Federation; countryCode: RU; stateProvince: Khanty-Mansiyskiy Avtonomnyy Okrug; county: Khanty-Mansiyskiy Rayon; locality: Mukhrino field station of YSU, 20 km SW from Khanty-Mansiysk; decimalLatitude: 60.892632; decimalLongitude: 68.677156; **Identification:** identifiedBy: Filippova, Nina|Zvyagina, Elena; dateIdentified: 2023-02-28; identificationRemarks: Identification based on morphological and molecular characters; **Event:** eventDate: 2013-09-11; habitat: Dwarfshrubs - sphagnum ombrotrophic bog

#### Cuphophyllus cinerellus

(Kühner) Bon

2E370B2D-9302-5351-930B-6ABBCD69B0A7

##### Materials

- **Type status:** Other material. **Occurrence:** catalogNumber: YSU-F-04491; recordedBy: Filippova, Nina; associatedSequences: OP866201; occurrenceID: 3F31C9B7-2A00-5889-967F-76ED1B1AFA7F; **Location:** country: Russian Federation; countryCode: RU; stateProvince: Khanty-Mansiyskiy Avtonomnyy Okrug; county: Khanty-Mansiyskiy Rayon; locality: Mukhrino field station of YSU, 20 km SW from Khanty-Mansiysk; decimalLatitude: 60.891777; decimalLongitude: 68.683476; **Identification:** identifiedBy: Filippova, Nina|Zvyagina, Elena; dateIdentified: 2023-02-28; identificationRemarks: Identification based on morphological and molecular characters; **Event:** eventDate: 2014-07-16; habitat: Andromeda-graminoid-S.papillosum lawn

#### Entoloma sp.

9EB27173-78FC-5F50-8DFC-0223526B82EC

##### Materials

- **Type status:** Other material. **Occurrence:** catalogNumber: YSU-F-12105; recordedBy: Filippova, Nina|Rudykina, Elena; associatedSequences: OQ396709; occurrenceID: 3CC67DC4-A033-5B55-98D1-5036492E873E; **Location:** country: Russian Federation; countryCode: RU; stateProvince: Khanty-Mansiyskiy Avtonomnyy Okrug; county: Khanty-Mansiyskiy Rayon; locality: Shapsha village vicinity, 20 km E from Khanty-Mansiysk; decimalLatitude: 61.066410; decimalLongitude: 69.468030; **Identification:** identifiedBy: Filippova, Nina|Zvyagina, Elena; dateIdentified: 2023-02-28; identificationRemarks: Identification based on morphological and molecular characters; **Event:** eventDate: 2022-07-30; habitat: Raised Sphagnum bog

#### Entoloma fuscomarginatum

P.D.Orton

1EBF862F-1EFC-5B08-BCAA-C9BDBC5BA230

##### Materials

- **Type status:** Other material. **Occurrence:** catalogNumber: YSU-F-10530; recordedBy: Filippova, Nina; associatedSequences: OP866236; occurrenceID: 6F67D9ED-1D19-5381-B253-FF0358B9D8CE; **Location:** country: Russian Federation; countryCode: RU; stateProvince: Khanty-Mansiyskiy Avtonomnyy Okrug; county: Khanty-Mansiyskiy Rayon; locality: Khanty-Mansiysk town vicinity; decimalLatitude: 60.891900; decimalLongitude: 68.682260; **Identification:** identifiedBy: Filippova, Nina|Zvyagina, Elena; dateIdentified: 2023-02-28; identificationRemarks: Identification based on morphological and molecular characters; **Event:** eventDate: 2020-09-08; habitat: Raised Sphagnum bog

#### Galerina allospora

A.H.Sm. & Singer

3C1B3E0C-80C7-579A-BF55-DAF09B23A65D

##### Materials

- **Type status:** Other material. **Occurrence:** catalogNumber: YSU-F-04385; recordedBy: Filippova, Nina; associatedSequences: OP866193; occurrenceID: 9F4E433F-0ACD-5C15-878A-03A54AC7AC4C; **Location:** country: Russian Federation; countryCode: RU; stateProvince: Khanty-Mansiyskiy Avtonomnyy Okrug; county: Khanty-Mansiyskiy Rayon; locality: Mukhrino field station of YSU, 20 km SW from Khanty-Mansiysk; decimalLatitude: 60.892632; decimalLongitude: 68.677156; **Identification:** identifiedBy: Filippova, Nina|Zvyagina, Elena; dateIdentified: 2023-02-28; identificationRemarks: Identification based on morphological and molecular characters; **Event:** eventDate: 2013-08-23; habitat: Dwarfshrubs - sphagnum ombrotrophic bog
- **Type status:** Other material. **Occurrence:** catalogNumber: YSU-F-05998; recordedBy: Filippova, Nina; associatedSequences: OP866210; occurrenceID: A9EB6A92-F709-590C-A139-B1995F9821B8; **Location:** country: Russian Federation; countryCode: RU; stateProvince: Khanty-Mansiyskiy Avtonomnyy Okrug; county: Khanty-Mansiyskiy Rayon; locality: Mukhrino field station of YSU, 20 km SW from Khanty-Mansiysk; decimalLatitude: 60.892010; decimalLongitude: 68.682420; **Identification:** identifiedBy: Filippova, Nina|Zvyagina, Elena; dateIdentified: 2023-02-28; identificationRemarks: Identification based on morphological and molecular characters; **Event:** eventDate: 2015-08-15; habitat: Pine - dwarfshrubs - S. fuscum ombrotrophic bog
- **Type status:** Other material. **Occurrence:** catalogNumber: YSU-F-06496; recordedBy: Filippova, Nina; associatedSequences: OQ380701; occurrenceID: D7CB0165-75E9-5CD8-BA5D-9D873324055C; **Location:** country: Russian Federation; countryCode: RU; stateProvince: Khanty-Mansiyskiy Avtonomnyy Okrug; county: Khanty-Mansiyskiy Rayon; locality: Mukhrino field station of YSU, 20 km SW from Khanty-Mansiysk; decimalLatitude: 60.892010; decimalLongitude: 68.682420; **Identification:** identifiedBy: Filippova, Nina|Zvyagina, Elena; dateIdentified: 2023-02-28; identificationRemarks: Identification based on morphological and molecular characters; **Event:** eventDate: 2015-09-05; habitat: Pine - dwarfshrubs - S. fuscum ombrotrophic bog
- **Type status:** Other material. **Occurrence:** catalogNumber: YSU-F-12176; recordedBy: Rudykina, Elena|Dobrynina, Alevtina; associatedSequences: OP866262; occurrenceID: 356B7B3C-6237-59B2-8197-4B69AAF9455E; **Location:** country: Russian Federation; countryCode: RU; stateProvince: Khanty-Mansiyskiy Avtonomnyy Okrug; county: Khanty-Mansiyskiy Rayon; locality: Mukhrino field station of YSU, 20 km SW from Khanty-Mansiysk; decimalLatitude: 60.891781; decimalLongitude: 68.684251; **Identification:** identifiedBy: Filippova, Nina|Zvyagina, Elena; dateIdentified: 2023-02-28; identificationRemarks: Identification based on morphological and molecular characters; **Event:** eventDate: 2022-08-19; habitat: Raised Sphagnum bog

#### Galerina calyptrata

P.D.Orton

B01AFDA2-C58B-545A-8F33-6630241A4135

##### Materials

- **Type status:** Other material. **Occurrence:** catalogNumber: YSU-F-04379; recordedBy: Filippova, Nina; associatedSequences: OQ380704; occurrenceID: D9071435-5E7C-5AC3-B060-7A5FF065DBF2; **Location:** country: Russian Federation; countryCode: RU; stateProvince: Khanty-Mansiyskiy Avtonomnyy Okrug; county: Khanty-Mansiyskiy Rayon; locality: Mukhrino field station of YSU, 20 km SW from Khanty-Mansiysk; decimalLatitude: 60.892632; decimalLongitude: 68.677156; **Identification:** identifiedBy: Filippova, Nina|Zvyagina, Elena; dateIdentified: 2023-02-28; identificationRemarks: Identification based on morphological and molecular characters; **Event:** eventDate: 2013-08-22; habitat: Dwarfshrubs - sphagnum ombrotrophic bog
- **Type status:** Other material. **Occurrence:** catalogNumber: YSU-F-03955; recordedBy: Filippova, Nina; associatedSequences: OQ380698; occurrenceID: 61C442AF-220C-5823-B953-6D23A88CAF0F; **Location:** country: Russian Federation; countryCode: RU; stateProvince: Khanty-Mansiyskiy Avtonomnyy Okrug; county: Khanty-Mansiyskiy Rayon; locality: Mukhrino field station of YSU, 20 km SW from Khanty-Mansiysk; decimalLatitude: 60.889934; decimalLongitude: 68.700686; **Identification:** identifiedBy: Filippova, Nina|Zvyagina, Elena; dateIdentified: 2023-02-28; identificationRemarks: Identification based on morphological and molecular characters; **Event:** eventDate: 2012-09-02; habitat: Pine - dwarfshrubs - sphagnum bog (close to forest)

#### Galerina paludosa

(Fr.) Kühner

A369E67D-8B90-50BD-B9E1-75B222E39087

##### Materials

- **Type status:** Other material. **Occurrence:** catalogNumber: YSU-F-12103; recordedBy: Filippova, Nina|Rudykina, Elena; associatedSequences: OP866242; occurrenceID: 76F13EF6-5A13-5249-914F-DF3161A2D8DC; **Location:** country: Russian Federation; countryCode: RU; stateProvince: Khanty-Mansiyskiy Avtonomnyy Okrug; county: Khanty-Mansiyskiy Rayon; locality: Shapsha village vicinity, 20 km E from Khanty-Mansiysk; decimalLatitude: 61.066410; decimalLongitude: 69.468030; **Identification:** identifiedBy: Filippova, Nina|Zvyagina, Elena; dateIdentified: 2023-02-28; identificationRemarks: Identification based on morphological and molecular characters; **Event:** eventDate: 2022-07-30; habitat: Raised Sphagnum bog

#### Galerina pumila

(Pers.) Singer

022F18B6-F64E-510A-AFF6-5BF521FD28C8

##### Materials

- **Type status:** Other material. **Occurrence:** catalogNumber: YSU-F-05827; recordedBy: Filippova, Nina; associatedSequences: OP866205; occurrenceID: C88B0D7E-A8C2-5598-B027-068560B9F88E; **Location:** country: Russian Federation; countryCode: RU; stateProvince: Khanty-Mansiyskiy Avtonomnyy Okrug; county: Khanty-Mansiyskiy Rayon; locality: Mukhrino field station of YSU, 20 km SW from Khanty-Mansiysk; decimalLatitude: 60.891781; decimalLongitude: 68.684251; **Identification:** identifiedBy: Filippova, Nina|Zvyagina, Elena; dateIdentified: 2023-02-28; identificationRemarks: Identification based on morphological and molecular characters; **Event:** eventDate: 2015-08-08; habitat: Pine - dwarfshrubs - S. fuscum ombrotrophic bog

#### Galerina sphagnicola

(G.F.Atk.) A.H.Sm. & Singer

22640BC8-68DD-5866-8A6A-D9092F6E3BC0

##### Materials

- **Type status:** Other material. **Occurrence:** catalogNumber: YSU-F-04084; recordedBy: Filippova, Nina; associatedSequences: OQ380703; occurrenceID: F4ADF2F4-A771-5298-9845-C767393CE0F8; **Location:** country: Russian Federation; countryCode: RU; stateProvince: Khanty-Mansiyskiy Avtonomnyy Okrug; county: Khanty-Mansiyskiy Rayon; locality: Mukhrino field station of YSU, 20 km SW from Khanty-Mansiysk; decimalLatitude: 60.893086; decimalLongitude: 68.677082; **Identification:** identifiedBy: Filippova, Nina|Zvyagina, Elena; dateIdentified: 2023-02-28; identificationRemarks: Identification based on morphological and molecular characters; **Event:** eventDate: 2012-09-09; habitat: Graminoid - sphagnum hollow (patterned ridge - hollow bog)
- **Type status:** Other material. **Occurrence:** catalogNumber: YSU-F-04399; recordedBy: Filippova, Nina; associatedSequences: OP866195; occurrenceID: 37ED525F-E7D0-5C77-83DB-9A176996BD73; **Location:** country: Russian Federation; countryCode: RU; stateProvince: Khanty-Mansiyskiy Avtonomnyy Okrug; county: Khanty-Mansiyskiy Rayon; locality: Mukhrino field station of YSU, 20 km SW from Khanty-Mansiysk; decimalLatitude: 60.892632; decimalLongitude: 68.677156; **Identification:** identifiedBy: Filippova, Nina|Zvyagina, Elena; dateIdentified: 2023-02-28; identificationRemarks: Identification based on morphological and molecular characters; **Event:** eventDate: 2013-08-26; habitat: Graminoid - sphagnum hollow (patterned ridge - hollow bog)
- **Type status:** Other material. **Occurrence:** catalogNumber: YSU-F-08473; recordedBy: Filippova, Nina; associatedSequences: OQ380702; occurrenceID: 08304FFF-8395-541F-97F6-9890B2C49A71; **Location:** country: Russian Federation; countryCode: RU; stateProvince: Tomskaya Oblast'; county: Tomskiy Rayon; locality: Orlovka village vicinity, Chernoye lake; decimalLatitude: 56.878320; decimalLongitude: 84.665770; **Identification:** identifiedBy: Filippova, Nina|Zvyagina, Elena; dateIdentified: 2023-02-28; identificationRemarks: Identification based on morphological and molecular characters; **Event:** eventDate: 2018-08-22; habitat: Raised Pine-dwarfshrubs-Sphagnum bog
- **Type status:** Other material. **Occurrence:** catalogNumber: YSU-F-12177; recordedBy: Rudykina, Elena|Dobrynina, Alevtina; associatedSequences: OP866263; occurrenceID: 981E7D40-5103-5120-BF9B-B205F715C140; **Location:** country: Russian Federation; countryCode: RU; stateProvince: Khanty-Mansiyskiy Avtonomnyy Okrug; county: Khanty-Mansiyskiy Rayon; locality: Mukhrino field station of YSU, 20 km SW from Khanty-Mansiysk; decimalLatitude: 60.891781; decimalLongitude: 68.684251; **Identification:** identifiedBy: Filippova, Nina|Zvyagina, Elena; dateIdentified: 2023-02-28; identificationRemarks: Identification based on morphological and molecular characters; **Event:** eventDate: 2022-08-19; habitat: Raised Sphagnum bog

#### Galerina tibiicystis

(G.F.Atk.) Kühner

86887303-DD9F-5241-B558-E292D185AB81

##### Materials

- **Type status:** Other material. **Occurrence:** catalogNumber: YSU-F-12123; recordedBy: Filippova, Nina|Rudykina, Elena; associatedSequences: OP866244; occurrenceID: 35ADD260-F108-547B-93A5-FD30D3737D5F; **Location:** country: Russian Federation; countryCode: RU; stateProvince: Khanty-Mansiyskiy Avtonomnyy Okrug; county: Khanty-Mansiyskiy Rayon; locality: Mukhrino field station of YSU, 20 km SW from Khanty-Mansiysk; decimalLatitude: 60.891781; decimalLongitude: 68.684251; **Identification:** identifiedBy: Filippova, Nina|Zvyagina, Elena; dateIdentified: 2023-02-28; identificationRemarks: Identification based on morphological and molecular characters; **Event:** eventDate: 2022-08-04; habitat: Raised Sphagnum bog
- **Type status:** Other material. **Occurrence:** catalogNumber: YSU-F-12104; recordedBy: Filippova, Nina|Rudykina, Elena; associatedSequences: OP866243; occurrenceID: 32F94061-10FC-5A75-BC59-531FDBE1264E; **Location:** country: Russian Federation; countryCode: RU; stateProvince: Khanty-Mansiyskiy Avtonomnyy Okrug; county: Khanty-Mansiyskiy Rayon; locality: Shapsha village vicinity, 20 km E from Khanty-Mansiysk; decimalLatitude: 61.066410; decimalLongitude: 69.468030; **Identification:** identifiedBy: Filippova, Nina|Zvyagina, Elena; dateIdentified: 2023-02-28; identificationRemarks: Identification based on morphological and molecular characters; **Event:** eventDate: 2022-07-30; habitat: Raised Sphagnum bog

#### Galerina sp.

89EAC93F-B899-5B1C-A0A1-C47A63970AC5

##### Materials

- **Type status:** Other material. **Occurrence:** catalogNumber: YSU-F-10529; recordedBy: Filippova, Nina; associatedSequences: OQ380700; occurrenceID: 951E5EAE-2A54-5883-B6B4-E90E4C7B0DD8; **Location:** country: Russian Federation; countryCode: RU; stateProvince: Khanty-Mansiyskiy Avtonomnyy Okrug; county: Khanty-Mansiyskiy Rayon; locality: Khanty-Mansiysk town vicinity; decimalLatitude: 60.891900; decimalLongitude: 68.682260; **Identification:** identifiedBy: Filippova, Nina|Zvyagina, Elena; dateIdentified: 2023-02-28; identificationRemarks: Identification based on morphological and molecular characters; **Event:** eventDate: 2020-09-08; habitat: Raised Sphagnum bog
- **Type status:** Other material. **Occurrence:** catalogNumber: YSU-F-08625; recordedBy: Filippova, Nina; associatedSequences: OQ380699; occurrenceID: E3DBA78B-FDAB-595B-BDF4-F95469711EFB; **Location:** country: Russian Federation; countryCode: RU; stateProvince: Khanty-Mansiyskiy Avtonomnyy Okrug; county: Khanty-Mansiyskiy Rayon; locality: Mukhrino field station of YSU, 20 km SW from Khanty-Mansiysk; decimalLatitude: 60.891781; decimalLongitude: 68.684251; **Identification:** identifiedBy: Filippova, Nina|Zvyagina, Elena; dateIdentified: 2023-02-28; identificationRemarks: Identification based on morphological and molecular characters; **Event:** eventDate: 2018-09-04; habitat: Raised Sphagnum bog

#### Galerina sp. 1

D71C7E06-4C1E-54D2-90D7-6F469C2C4405

##### Materials

- **Type status:** Other material. **Occurrence:** catalogNumber: YSU-F-07835; recordedBy: Filippova, Nina; associatedSequences: OQ380706; occurrenceID: CA77951C-ECB8-5423-8A81-76E9A809FED4; **Location:** country: Russian Federation; countryCode: RU; stateProvince: Khanty-Mansiyskiy Avtonomnyy Okrug; county: Khanty-Mansiyskiy Rayon; locality: Mukhrino field station of YSU, 20 km SW from Khanty-Mansiysk; decimalLatitude: 60.891781; decimalLongitude: 68.684251; **Identification:** identifiedBy: Filippova, Nina|Zvyagina, Elena; dateIdentified: 2023-02-28; identificationRemarks: Identification based on morphological and molecular characters; **Event:** eventDate: 2017-07-08; habitat: Pine-dwarfshrubs-S.fuscum ombrotrophic bog
- **Type status:** Other material. **Occurrence:** catalogNumber: YSU-F-12130; recordedBy: Filippova, Nina|Rudykina, Elena; associatedSequences: OQ380707; occurrenceID: 3C821BD6-E7CD-55A2-9F7C-ECE6FCBCA496; **Location:** country: Russian Federation; countryCode: RU; stateProvince: Khanty-Mansiyskiy Avtonomnyy Okrug; county: Khanty-Mansiyskiy Rayon; locality: Mukhrino field station of YSU, 20 km SW from Khanty-Mansiysk; decimalLatitude: 60.891781; decimalLongitude: 68.684251; **Identification:** identifiedBy: Filippova, Nina|Zvyagina, Elena; dateIdentified: 2023-02-28; identificationRemarks: Identification based on morphological and molecular characters; **Event:** eventDate: 2022-08-04; habitat: Raised Sphagnum bog
- **Type status:** Other material. **Occurrence:** catalogNumber: YSU-F-12175; recordedBy: Rudykina, Elena|Dobrynina, Alevtina; associatedSequences: OQ380708; occurrenceID: 12D9BFB3-F636-55CD-8B75-5E847BB70E10; **Location:** country: Russian Federation; countryCode: RU; stateProvince: Khanty-Mansiyskiy Avtonomnyy Okrug; county: Khanty-Mansiyskiy Rayon; locality: Mukhrino field station of YSU, 20 km SW from Khanty-Mansiysk; decimalLatitude: 60.891781; decimalLongitude: 68.684251; **Identification:** identifiedBy: Filippova, Nina|Zvyagina, Elena; dateIdentified: 2023-02-28; identificationRemarks: Identification based on morphological and molecular characters; **Event:** eventDate: 2022-08-19; habitat: Raised Sphagnum bog
- **Type status:** Other material. **Occurrence:** catalogNumber: YSU-F-05828; recordedBy: Filippova, Nina; associatedSequences: OQ380705; occurrenceID: 2B917C8B-A819-5FE6-886B-81C1E6BF2BFF; **Location:** country: Russian Federation; countryCode: RU; stateProvince: Khanty-Mansiyskiy Avtonomnyy Okrug; county: Khanty-Mansiyskiy Rayon; locality: Mukhrino field station of YSU, 20 km SW from Khanty-Mansiysk; decimalLatitude: 60.891781; decimalLongitude: 68.684251; **Identification:** identifiedBy: Filippova, Nina|Zvyagina, Elena; dateIdentified: 2023-02-28; identificationRemarks: Identification based on morphological and molecular characters; **Event:** eventDate: 2015-08-08; habitat: Pine - dwarfshrubs - S. fuscum ombrotrophic bog

#### Galerina sp. 2

FCE96BD9-947B-557D-8789-3F85604E6E7E

##### Materials

- **Type status:** Other material. **Occurrence:** catalogNumber: YSU-F-08626; recordedBy: Filippova, Nina; associatedSequences: OQ380709; occurrenceID: 53F60077-A223-5EDD-907F-027ECE8F82CA; **Location:** country: Russian Federation; countryCode: RU; stateProvince: Khanty-Mansiyskiy Avtonomnyy Okrug; county: Khanty-Mansiyskiy Rayon; locality: Mukhrino field station of YSU, 20 km SW from Khanty-Mansiysk; decimalLatitude: 60.891781; decimalLongitude: 68.684251; **Identification:** identifiedBy: Filippova, Nina|Zvyagina, Elena; dateIdentified: 2023-02-28; identificationRemarks: Identification based on morphological and molecular characters; **Event:** eventDate: 2018-09-04; habitat: Raised Sphagnum bog

#### Gymnopilus decipiens

(Sacc.) P.D.Orton

10270D09-88E1-5AE8-8201-6432EF16B9A9

##### Materials

- **Type status:** Other material. **Occurrence:** catalogNumber: YSU-F-10528; recordedBy: Filippova, Nina; associatedSequences: OP866235; occurrenceID: C1484C7B-48C6-52A3-B90F-4EA4F07B1B13; **Location:** country: Russian Federation; countryCode: RU; stateProvince: Khanty-Mansiyskiy Avtonomnyy Okrug; county: Khanty-Mansiyskiy Rayon; locality: Khanty-Mansiysk town vicinity; decimalLatitude: 60.891900; decimalLongitude: 68.682260; **Identification:** identifiedBy: Filippova, Nina|Zvyagina, Elena; dateIdentified: 2023-02-28; identificationRemarks: Identification based on morphological and molecular characters; **Event:** eventDate: 2020-09-08; habitat: Raised Sphagnum bog
- **Type status:** Other material. **Occurrence:** catalogNumber: YSU-F-06667; recordedBy: Filippova, Nina; associatedSequences: OP866215; occurrenceID: F20A3E45-F70F-5E8C-B9EA-E3784475243C; **Location:** country: Russian Federation; countryCode: RU; stateProvince: Khanty-Mansiyskiy Avtonomnyy Okrug; county: Khanty-Mansiyskiy Rayon; locality: Mukhrino field station of YSU, 20 km SW from Khanty-Mansiysk; decimalLatitude: 60.891781; decimalLongitude: 68.684251; **Identification:** identifiedBy: Filippova, Nina|Zvyagina, Elena; dateIdentified: 2023-02-28; identificationRemarks: Identification based on morphological and molecular characters; **Event:** eventDate: 2016-07-02; habitat: Treed pine-dwarfshrubs-S. fuscum ombrotrophic bog
- **Type status:** Other material. **Occurrence:** catalogNumber: YSU-F-12129; recordedBy: Filippova, Nina|Rudykina, Elena; associatedSequences: OP866249; occurrenceID: 040F08EB-00DE-5047-99B3-84EB81A2D5C4; **Location:** country: Russian Federation; countryCode: RU; stateProvince: Khanty-Mansiyskiy Avtonomnyy Okrug; county: Khanty-Mansiyskiy Rayon; locality: Mukhrino field station of YSU, 20 km SW from Khanty-Mansiysk; decimalLatitude: 60.891781; decimalLongitude: 68.684251; **Identification:** identifiedBy: Filippova, Nina|Zvyagina, Elena; dateIdentified: 2023-02-28; identificationRemarks: Identification based on morphological and molecular characters; **Event:** eventDate: 2022-08-04; habitat: Raised Sphagnum bog

#### Gymnopilus sp.

EC450C09-3A18-51BB-A319-F97A7502F82C

##### Materials

- **Type status:** Other material. **Occurrence:** catalogNumber: YSU-F-04064; recordedBy: Filippova, Nina; associatedSequences: OQ380715; occurrenceID: 0617518F-761D-559E-840F-B7C675AE8556; **Location:** country: Russian Federation; countryCode: RU; stateProvince: Khanty-Mansiyskiy Avtonomnyy Okrug; county: Khanty-Mansiyskiy Rayon; locality: Mukhrino field station of YSU, 20 km SW from Khanty-Mansiysk; decimalLatitude: 60.888786; decimalLongitude: 68.686395; **Identification:** identifiedBy: Filippova, Nina|Zvyagina, Elena; dateIdentified: 2023-02-28; identificationRemarks: Identification based on morphological and molecular characters; **Event:** eventDate: 2012-09-08; habitat: Graminoid - sphagnum hollow (patterned ridge - hollow bog)
- **Type status:** Other material. **Occurrence:** catalogNumber: YSU-F-08623; recordedBy: Filippova, Nina; associatedSequences: OQ406271; occurrenceID: 2687BE71-3B7A-5A5D-A458-AF2A3B328677; **Location:** country: Russian Federation; countryCode: RU; stateProvince: Khanty-Mansiyskiy Avtonomnyy Okrug; county: Khanty-Mansiyskiy Rayon; locality: Mukhrino field station of YSU, 20 km SW from Khanty-Mansiysk; decimalLatitude: 60.891781; decimalLongitude: 68.684251; **Identification:** identifiedBy: Filippova, Nina|Zvyagina, Elena; dateIdentified: 2023-02-28; identificationRemarks: Identification based on morphological and molecular characters; **Event:** eventDate: 2018-09-04; habitat: Raised Sphagnum bog

#### Gymnopus androsaceus

(L.) Della Magg. & Trassin.

66DDED05-3812-5BEC-A55F-E2B3F4913593

##### Materials

- **Type status:** Other material. **Occurrence:** catalogNumber: YSU-F-04985; recordedBy: Filippova, Nina; associatedSequences: OP866203; occurrenceID: 54B1176F-120F-528A-B1B1-F64B28894464; **Location:** country: Russian Federation; countryCode: RU; stateProvince: Khanty-Mansiyskiy Avtonomnyy Okrug; county: Khanty-Mansiyskiy Rayon; locality: Mukhrino field station of YSU, 20 km SW from Khanty-Mansiysk; decimalLatitude: 60.891980; decimalLongitude: 68.682430; **Identification:** identifiedBy: Filippova, Nina|Zvyagina, Elena; dateIdentified: 2023-02-28; identificationRemarks: Identification based on morphological and molecular characters; **Event:** eventDate: 2015-06-06; habitat: Pine - dwarfshrubs - S. fuscum ombrotrophic bog

#### Gymnopus dryophilus

(Bull.) Murrill

3D36970D-0D92-576B-9107-0AC703B0E23E

##### Materials

- **Type status:** Other material. **Occurrence:** catalogNumber: YSU-F-09780; recordedBy: Filippova, Nina; associatedSequences: OQ366384; occurrenceID: D4246552-F6B7-5E71-BEC1-3FD211BAC035; **Location:** country: Russian Federation; countryCode: RU; stateProvince: Khanty-Mansiyskiy Avtonomnyy Okrug; county: Khanty-Mansiyskiy Rayon; locality: Khanty-Mansiysk town vicinity; decimalLatitude: 60.89171; decimalLongitude: 68.68451; **Identification:** identifiedBy: Filippova, Nina|Zvyagina, Elena; dateIdentified: 2023-02-28; identificationRemarks: Identification based on morphological and molecular characters; **Event:** eventDate: 2020-06-28; habitat: Raised bog with Pinussylvestris

#### Gymnopus junquilleus

R.H.Petersen & J.L.Mata

DF736571-C9A4-523E-8E3F-66F17920FB83

##### Materials

- **Type status:** Other material. **Occurrence:** catalogNumber: YSU-F-04971; recordedBy: Filippova, Nina; associatedSequences: OP866202; occurrenceID: 0C7C3986-A599-52D6-8600-0939AB20202E; **Location:** country: Russian Federation; countryCode: RU; stateProvince: Khanty-Mansiyskiy Avtonomnyy Okrug; county: Khanty-Mansiyskiy Rayon; locality: Mukhrino field station of YSU, 20 km SW from Khanty-Mansiysk; decimalLatitude: 60.891980; decimalLongitude: 68.682430; **Identification:** identifiedBy: Filippova, Nina|Zvyagina, Elena; dateIdentified: 2023-02-28; identificationRemarks: Identification based on morphological and molecular characters; **Event:** eventDate: 2015-05-31; habitat: Pine - dwarfshrubs - S. fuscum ombrotrophic bog
- **Type status:** Other material. **Occurrence:** catalogNumber: YSU-F-07912; recordedBy: Filippova, Nina; associatedSequences: OQ366385; occurrenceID: 456E3810-0F1B-584A-B4CE-247A84745570; **Location:** country: Russian Federation; countryCode: RU; stateProvince: Khanty-Mansiyskiy Avtonomnyy Okrug; county: Khanty-Mansiyskiy Rayon; locality: Mukhrino field station of YSU, 20 km SW from Khanty-Mansiysk; decimalLatitude: 60.891781; decimalLongitude: 68.684251; **Identification:** identifiedBy: Filippova, Nina|Zvyagina, Elena; dateIdentified: 2023-02-28; identificationRemarks: Identification based on morphological and molecular characters; **Event:** eventDate: 2017-08-07; habitat: Pine-dwarfshrubs-S.fuscum ombrotrophic bog
- **Type status:** Other material. **Occurrence:** catalogNumber: YSU-F-08014; recordedBy: Filippova, Nina; associatedSequences: OP866220; occurrenceID: 98E9EC09-FFB1-5D33-A2C0-1967413DFF9A; **Location:** country: Russian Federation; countryCode: RU; stateProvince: Khanty-Mansiyskiy Avtonomnyy Okrug; county: Khanty-Mansiyskiy Rayon; locality: Mukhrino field station of YSU, 20 km SW from Khanty-Mansiysk; decimalLatitude: 60.891954; decimalLongitude: 68.687647; **Identification:** identifiedBy: Filippova, Nina|Zvyagina, Elena; dateIdentified: 2023-02-28; identificationRemarks: Identification based on morphological and molecular characters; **Event:** eventDate: 2018-06-24; habitat: Raised bog, among S. fuscum

#### Gymnopus sp.

986A8DEC-65A0-590A-9793-0AB9ACB4D978

##### Materials

- **Type status:** Other material. **Occurrence:** catalogNumber: YSU-F-06283; recordedBy: Filippova, Nina; associatedSequences: OQ366383; occurrenceID: 29EBDEB9-670A-5D90-BAD5-F374FA3C9141; **Location:** country: Russian Federation; countryCode: RU; stateProvince: Khanty-Mansiyskiy Avtonomnyy Okrug; county: Khanty-Mansiyskiy Rayon; locality: Mukhrino field station of YSU, 20 km SW from Khanty-Mansiysk; decimalLatitude: 60.892010; decimalLongitude: 68.682420; **Identification:** identifiedBy: Filippova, Nina|Zvyagina, Elena; dateIdentified: 2023-02-28; identificationRemarks: Identification based on morphological and molecular characters; **Event:** eventDate: 2015-08-24; habitat: Pine - dwarfshrubs - S. fuscum ombrotrophic bog
- **Type status:** Other material. **Occurrence:** catalogNumber: YSU-F-05835; recordedBy: Filippova, Nina; associatedSequences: OQ366382; occurrenceID: 61A8E942-28C0-5A9A-824D-91291C25A7C0; **Location:** country: Russian Federation; countryCode: RU; stateProvince: Khanty-Mansiyskiy Avtonomnyy Okrug; county: Khanty-Mansiyskiy Rayon; locality: Mukhrino field station of YSU, 20 km SW from Khanty-Mansiysk; decimalLatitude: 60.891781; decimalLongitude: 68.684251; **Identification:** identifiedBy: Filippova, Nina|Zvyagina, Elena; dateIdentified: 2023-02-28; identificationRemarks: Identification based on morphological and molecular characters; **Event:** eventDate: 2015-08-08; habitat: Pine - dwarfshrubs - S. fuscum ombrotrophic bog
- **Type status:** Other material. **Occurrence:** catalogNumber: YSU-F-03953; recordedBy: Filippova, Nina; associatedSequences: OQ406264; occurrenceID: 27064764-4968-5BFA-ADFA-E7F927D79440; **Location:** country: Russian Federation; countryCode: RU; stateProvince: Khanty-Mansiyskiy Avtonomnyy Okrug; county: Khanty-Mansiyskiy Rayon; locality: Mukhrino field station of YSU, 20 km SW from Khanty-Mansiysk; decimalLatitude: 60.889934; decimalLongitude: 68.700686; **Identification:** identifiedBy: Filippova, Nina|Zvyagina, Elena; dateIdentified: 2023-02-28; identificationRemarks: Identification based on morphological and molecular characters; **Event:** eventDate: 2012-09-02; habitat: Pine - dwarfshrubs - sphagnum bog (close to forest)

#### Gyroflexus brevibasidiatus

(Singer) Redhead, Moncalvo, Vilgalys & Lutzoni

6AA4DCE7-9612-5375-883E-4D7C88E9A23D

##### Materials

- **Type status:** Other material. **Occurrence:** catalogNumber: YSU-F-05832; recordedBy: Filippova, Nina; associatedSequences: OP866206; occurrenceID: 239FF4B4-CF6B-5A88-AB33-6D96E083D365; **Location:** country: Russian Federation; countryCode: RU; stateProvince: Khanty-Mansiyskiy Avtonomnyy Okrug; county: Khanty-Mansiyskiy Rayon; locality: Mukhrino field station of YSU, 20 km SW from Khanty-Mansiysk; decimalLatitude: 60.891781; decimalLongitude: 68.684251; **Identification:** identifiedBy: Filippova, Nina|Zvyagina, Elena; dateIdentified: 2023-02-28; identificationRemarks: Identification based on morphological and molecular characters; **Event:** eventDate: 2015-08-08; habitat: Pine - dwarfshrubs - S. fuscum ombrotrophic bog
- **Type status:** Other material. **Occurrence:** catalogNumber: YSU-F-12127; recordedBy: Filippova, Nina|Rudykina, Elena; associatedSequences: OP866248; occurrenceID: BBC81A3F-DD66-5775-A3E2-9519F52F52DA; **Location:** country: Russian Federation; countryCode: RU; stateProvince: Khanty-Mansiyskiy Avtonomnyy Okrug; county: Khanty-Mansiyskiy Rayon; locality: Mukhrino field station of YSU, 20 km SW from Khanty-Mansiysk; decimalLatitude: 60.891781; decimalLongitude: 68.684251; **Identification:** identifiedBy: Filippova, Nina|Zvyagina, Elena; dateIdentified: 2023-02-28; identificationRemarks: Identification based on morphological and molecular characters; **Event:** eventDate: 2022-08-04; habitat: Raised Sphagnum bog

#### Hebeloma incarnatulum

A.H.Sm.

A522DBCE-29EE-5B93-98CC-5C93368D90A2

##### Materials

- **Type status:** Other material. **Occurrence:** catalogNumber: YSU-F-04390; recordedBy: Filippova, Nina; associatedSequences: OP866194; occurrenceID: 10DE3EFF-6D58-56BA-9515-1DFDBA7413AF; **Location:** country: Russian Federation; countryCode: RU; stateProvince: Khanty-Mansiyskiy Avtonomnyy Okrug; county: Khanty-Mansiyskiy Rayon; locality: Mukhrino field station of YSU, 20 km SW from Khanty-Mansiysk; decimalLatitude: 60.892632; decimalLongitude: 68.677156; **Identification:** identifiedBy: Filippova, Nina|Zvyagina, Elena; dateIdentified: 2023-02-28; identificationRemarks: Identification based on morphological and molecular characters; **Event:** eventDate: 2013-08-24; habitat: Dwarfshrubs - sphagnum ombrotrophic bog
- **Type status:** Other material. **Occurrence:** catalogNumber: YSU-F-03939; recordedBy: Filippova, Nina; associatedSequences: OP866184; occurrenceID: B7D02454-1B7E-5C61-9769-1501A485CEFA; **Location:** country: Russian Federation; countryCode: RU; stateProvince: Khanty-Mansiyskiy Avtonomnyy Okrug; county: Khanty-Mansiyskiy Rayon; locality: Mukhrino field station of YSU, 20 km SW from Khanty-Mansiysk; decimalLatitude: 60.889934; decimalLongitude: 68.700686; **Identification:** identifiedBy: Filippova, Nina|Zvyagina, Elena; dateIdentified: 2023-02-28; identificationRemarks: Identification based on morphological and molecular characters; **Event:** eventDate: 2012-09-01; habitat: Pine - dwarfshrubs - sphagnum bog (close to forest)

#### Hypholoma capnoides

(Fr.) P.Kumm.

5E33EA86-27F1-59C8-96A7-9CCF421DAADE

##### Materials

- **Type status:** Other material. **Occurrence:** recordedBy: Filippova, Nina; occurrenceID: C1D32E79-F1F2-51A2-9573-E7B6B0D4405D; **Location:** country: Russian Federation; countryCode: RU; county: Khanty-Mansiyskiy Rayon; locality: Mukhrino field station of YSU, 20 km SW from Khanty-Mansiysk; decimalLatitude: 60.891781; decimalLongitude: 68.684251; **Identification:** identifiedBy: Filippova, Nina; identificationRemarks: Identification based on observation, no collections were made; **Event:** eventDate: 2022-08-19; habitat: Raised Sphagnum bog

#### Hypholoma elongatum

(Pers.) Ricken

D7EE7BC9-3A8E-5595-A730-694D2D91990D

##### Materials

- **Type status:** Other material. **Occurrence:** catalogNumber: YSU-F-07318; recordedBy: Filippova, Nina; associatedSequences: OP866218; occurrenceID: C9408C4B-5F38-5851-861E-113D1435D4E8; **Location:** country: Russian Federation; countryCode: RU; stateProvince: Khanty-Mansiyskiy Avtonomnyy Okrug; county: Khanty-Mansiyskiy Rayon; locality: Mukhrino field station of YSU, 20 km SW from Khanty-Mansiysk; decimalLatitude: 60.891781; decimalLongitude: 68.684251; **Identification:** identifiedBy: Filippova, Nina|Zvyagina, Elena; dateIdentified: 2023-02-28; identificationRemarks: Identification based on morphological and molecular characters; **Event:** eventDate: 2016-09-05; habitat: Graminoid-Sphagnum ombrotrophic hollow
- **Type status:** Other material. **Occurrence:** catalogNumber: YSU-F-04112; recordedBy: Filippova, Nina; associatedSequences: OP866192; occurrenceID: CCAF12D1-ADEC-5BBF-87D5-48E3D3BC3AD4; **Location:** country: Russian Federation; countryCode: RU; stateProvince: Khanty-Mansiyskiy Avtonomnyy Okrug; county: Khanty-Mansiyskiy Rayon; locality: Mukhrino field station of YSU, 20 km SW from Khanty-Mansiysk; decimalLatitude: 60.900413; decimalLongitude: 68.691845; **Identification:** identifiedBy: Filippova, Nina|Zvyagina, Elena; dateIdentified: 2023-02-28; identificationRemarks: Identification based on morphological and molecular characters; **Event:** eventDate: 2012-09-11; habitat: Pine - dwarfshrubs - sphagnum bog (close to forest)

#### Laccaria proxima

(Boud.) Pat.

4C6341FC-A8E1-5145-9C69-9037917722BC

##### Materials

- **Type status:** Other material. **Occurrence:** catalogNumber: YSU-F-04431; recordedBy: Filippova, Nina; associatedSequences: OP866199; occurrenceID: C0716471-51D7-56AF-9105-62D8314553D6; **Location:** country: Russian Federation; countryCode: RU; stateProvince: Khanty-Mansiyskiy Avtonomnyy Okrug; county: Khanty-Mansiyskiy Rayon; locality: Mukhrino field station of YSU, 20 km SW from Khanty-Mansiysk; decimalLatitude: 60.892632; decimalLongitude: 68.677156; **Identification:** identifiedBy: Filippova, Nina|Zvyagina, Elena; dateIdentified: 2023-02-28; identificationRemarks: Identification based on morphological and molecular characters; **Event:** eventDate: 2013-09-15; habitat: Dwarfshrubs - sphagnum ombrotrophic bog

#### Lactarius helvus

(Fr.) Fr.

AAA618C4-8DE6-5CEA-9CF3-56065FA6D281

##### Materials

- **Type status:** Other material. **Occurrence:** catalogNumber: YSU-F-04428; recordedBy: Filippova, Nina; associatedSequences: OP866198; occurrenceID: 63925012-C9DD-5F8E-9885-409B28E21462; **Location:** country: Russian Federation; countryCode: RU; stateProvince: Khanty-Mansiyskiy Avtonomnyy Okrug; county: Khanty-Mansiyskiy Rayon; locality: Mukhrino field station of YSU, 20 km SW from Khanty-Mansiysk; decimalLatitude: 60.892632; decimalLongitude: 68.677156; **Identification:** identifiedBy: Filippova, Nina|Zvyagina, Elena; dateIdentified: 2023-02-28; identificationRemarks: Identification based on morphological and molecular characters; **Event:** eventDate: 2013-09-14; habitat: Dwarfshrubs - sphagnum ombrotrophic bog

#### Lactarius musteus

Fr.

5E34E94B-54B8-5CA4-920B-B98AF0F2C397

##### Materials

- **Type status:** Other material. **Occurrence:** catalogNumber: YSU-F-08240; recordedBy: Filippova, Nina|Tomkovich, Konstantin; associatedSequences: OP866222; occurrenceID: 9C437F0F-6A05-59B1-9FBC-7CC78BF9DD07; **Location:** country: Russian Federation; countryCode: RU; stateProvince: Khanty-Mansiyskiy Avtonomnyy Okrug; county: Khanty-Mansiyskiy Rayon; locality: Shapsha village vicinity, 20 km E from Khanty-Mansiysk; decimalLatitude: 61.062728; decimalLongitude: 69.478030; **Identification:** identifiedBy: Filippova, Nina|Zvyagina, Elena; dateIdentified: 2023-02-28; identificationRemarks: Identification based on morphological and molecular characters; **Event:** eventDate: 2018-08-05; habitat: Treed bog, "tall ryam"
- **Type status:** Other material. **Occurrence:** catalogNumber: YSU-F-12126; recordedBy: Filippova, Nina|Rudykina, Elena; associatedSequences: OP866247; occurrenceID: A6F1DF16-F341-51CA-B346-BEF5B9E05631; **Location:** country: Russian Federation; countryCode: RU; stateProvince: Khanty-Mansiyskiy Avtonomnyy Okrug; county: Khanty-Mansiyskiy Rayon; locality: Mukhrino field station of YSU, 20 km SW from Khanty-Mansiysk; decimalLatitude: 60.891781; decimalLongitude: 68.684251; **Identification:** identifiedBy: Filippova, Nina|Zvyagina, Elena; dateIdentified: 2023-02-28; identificationRemarks: Identification based on morphological and molecular characters; **Event:** eventDate: 2022-08-04; habitat: Raised Sphagnum bog

#### Lactarius rufus

(Scop.) Fr.

4FF1F82D-F2DC-5E7C-8D66-AFC91D85BBB9

##### Materials

- **Type status:** Other material. **Occurrence:** catalogNumber: YSU-F-09780; recordedBy: Filippova, Nina; associatedSequences: OQ366384; occurrenceID: AFAAC398-7421-5C6F-9508-FC4102D17FED; **Location:** country: Russian Federation; countryCode: RU; stateProvince: Khanty-Mansiyskiy Avtonomnyy Okrug; county: Khanty-Mansiyskiy Rayon; locality: Khanty-Mansiysk town vicinity; decimalLatitude: 60.891710; decimalLongitude: 68.684510; **Identification:** identifiedBy: Filippova, Nina|Zvyagina, Elena; dateIdentified: 2023-02-28; identificationRemarks: Identification based on morphological and molecular characters; **Event:** eventDate: 2020-06-28; habitat: Raised bog with Pinussylvestris
- **Type status:** Other material. **Occurrence:** catalogNumber: YSU-F-12133; recordedBy: Filippova, Nina|Rudykina, Elena; associatedSequences: OP866251; occurrenceID: E7B52401-F6D5-5FD2-9198-1DE6144A80D9; **Location:** country: Russian Federation; countryCode: RU; stateProvince: Khanty-Mansiyskiy Avtonomnyy Okrug; county: Khanty-Mansiyskiy Rayon; locality: Mukhrino field station of YSU, 20 km SW from Khanty-Mansiysk; decimalLatitude: 60.891781; decimalLongitude: 68.684251; **Identification:** identifiedBy: Filippova, Nina|Zvyagina, Elena; dateIdentified: 2023-02-28; identificationRemarks: Identification based on morphological and molecular characters; **Event:** eventDate: 2022-08-04; habitat: Raised Sphagnum bog

#### Lactarius uvidus

(Fr.) Fr.

EA8C486D-F5FC-53B8-B2E4-89BECA211C84

##### Materials

- **Type status:** Other material. **Occurrence:** recordedBy: Filippova, Nina; occurrenceID: 90161AAA-08CF-5D8F-A5D2-555B537BFEBC; **Location:** country: Russian Federation; countryCode: RU; county: Khanty-Mansiyskiy Rayon; locality: Mukhrino field station of YSU, 20 km SW from Khanty-Mansiysk; decimalLatitude: 60.891781; decimalLongitude: 68.684251; **Identification:** identifiedBy: Filippova, Nina; identificationRemarks: Identification based on observation, no collections were made; **Event:** eventDate: 2022-08-19; habitat: Raised Sphagnum bog

#### Lactarius vietus

(Fr.) Fr.

91B4488C-6A11-57C0-A330-E5671EFC8613

##### Materials

- **Type status:** Other material. **Occurrence:** catalogNumber: YSU-F-12178; recordedBy: Rudykina, Elena|Dobrynina, Alevtina; associatedSequences: OP866264; occurrenceID: 26CE9CCD-09B5-57F1-83BA-5B76491EBE6E; **Location:** country: Russian Federation; countryCode: RU; stateProvince: Khanty-Mansiyskiy Avtonomnyy Okrug; county: Khanty-Mansiyskiy Rayon; locality: Mukhrino field station of YSU, 20 km SW from Khanty-Mansiysk; decimalLatitude: 60.891781; decimalLongitude: 68.684251; **Identification:** identifiedBy: Filippova, Nina|Zvyagina, Elena; dateIdentified: 2023-02-28; identificationRemarks: Identification based on morphological and molecular characters; **Event:** eventDate: 2022-08-19; habitat: Raised Sphagnum bog

#### Leccinum holopus

(Rostk.) Watling

9E869D96-4AB0-5E4F-A829-D6DE644D0974

##### Materials

- **Type status:** Other material. **Occurrence:** catalogNumber: YSU-F-12179; recordedBy: Rudykina, Elena|Dobrynina, Alevtina; associatedSequences: OQ406273; occurrenceID: 349038D6-D530-545A-B837-9529CAFA898D; **Location:** country: Russian Federation; countryCode: RU; stateProvince: Khanty-Mansiyskiy Avtonomnyy Okrug; county: Khanty-Mansiyskiy Rayon; locality: Mukhrino field station of YSU, 20 km SW from Khanty-Mansiysk; decimalLatitude: 60.891781; decimalLongitude: 68.684251; **Identification:** identifiedBy: Filippova, Nina|Zvyagina, Elena; dateIdentified: 2023-02-28; identificationRemarks: Identification based on morphological and molecular characters; **Event:** eventDate: 2022-08-19; habitat: Raised Sphagnum bog

#### Leccinum schistophilum

Bon

07FAA650-AA5E-5971-AB8E-E2F7195A4DD7

##### Materials

- **Type status:** Other material. **Occurrence:** catalogNumber: YSU-F-12102; recordedBy: Filippova, Nina; associatedSequences: OP866241; occurrenceID: CDAACA96-1B15-528D-8CF1-570D46A65DD5; **Location:** country: Russian Federation; countryCode: RU; stateProvince: Khanty-Mansiyskiy Avtonomnyy Okrug; county: Khanty-Mansiyskiy Rayon; locality: Shapsha village vicinity, 20 km E from Khanty-Mansiysk; decimalLatitude: 61.066410; decimalLongitude: 69.468030; **Identification:** identifiedBy: Filippova, Nina|Zvyagina, Elena; dateIdentified: 2023-02-28; identificationRemarks: Identification based on morphological and molecular characters; **Event:** eventDate: 2022-07-30; habitat: Raised Sphagnum bog

#### Lichenomphalia umbellifera

(L.) Redhead, Lutzoni, Moncalvo & Vilgalys

A0CAA578-0770-5875-A1C8-7FD8FFA28C48

##### Materials

- **Type status:** Other material. **Occurrence:** catalogNumber: YSU-F-05069; recordedBy: Filippova, Nina; associatedSequences: OP866204; occurrenceID: 1CE95FFA-FC4C-5032-BC7C-2D21972CA400; **Location:** country: Russian Federation; countryCode: RU; stateProvince: Khanty-Mansiyskiy Avtonomnyy Okrug; county: Khanty-Mansiyskiy Rayon; locality: Mukhrino field station of YSU, 20 km SW from Khanty-Mansiysk; decimalLatitude: 60.888930; decimalLongitude: 68.702550; **Identification:** identifiedBy: Filippova, Nina|Zvyagina, Elena; dateIdentified: 2023-02-28; identificationRemarks: Identification based on morphological and molecular characters; **Event:** eventDate: 2015-06-20; habitat: Pine - dwarfshrubs - S. fuscum ombrotrophic bog

#### Mycena sp.

DCF1071E-27A4-5855-8591-2BBA55B740A5

##### Materials

- **Type status:** Other material. **Occurrence:** catalogNumber: YSU-F-03932; recordedBy: Filippova, Nina; associatedSequences: OQ407681; occurrenceID: 25EBDDF2-099A-5EC8-9CE3-9B943C809129; **Location:** country: Russian Federation; countryCode: RU; stateProvince: Khanty-Mansiyskiy Avtonomnyy Okrug; county: Khanty-Mansiyskiy Rayon; locality: Mukhrino field station of YSU, 20 km SW from Khanty-Mansiysk; decimalLatitude: 60.892773; decimalLongitude: 68.674893; **Identification:** identifiedBy: Filippova, Nina|Zvyagina, Elena; dateIdentified: 2023-02-28; identificationRemarks: Identification based on morphological and molecular characters; **Event:** eventDate: 2012-09-02; habitat: Pine - dwarfshrubs - sphagnum bog (close to forest)

#### Mycena galopus

(Pers.) P.Kumm.

CB4F39CE-5CCA-5AF4-A9C0-7765245E6EE5

##### Materials

- **Type status:** Other material. **Occurrence:** catalogNumber: YSU-F-03777; recordedBy: Filippova, Nina; associatedSequences: OQ407680; occurrenceID: 793379D3-298C-533F-8196-2DADA32369D5; **Location:** country: Russian Federation; countryCode: RU; stateProvince: Khanty-Mansiyskiy Avtonomnyy Okrug; county: Khanty-Mansiyskiy Rayon; locality: Chistoe bog, 20 km E from Khanty-Mansiysk; decimalLatitude: 61.064432; decimalLongitude: 69.460545; **Identification:** identifiedBy: Filippova, Nina|Zvyagina, Elena; dateIdentified: 2023-02-28; identificationRemarks: Identification based on morphological and molecular characters; **Event:** eventDate: 2012-08-18; habitat: Dwarfshrubs - sphagnum ombrotrophic bog

#### Mycena megaspora

Kauffman

3FE59BB9-B3D9-5EE5-99FF-7AB60954FBCB

##### Materials

- **Type status:** Other material. **Occurrence:** catalogNumber: YSU-F-12174; recordedBy: Rudykina, Elena|Dobrynina, Alevtina; associatedSequences: OP866261; occurrenceID: EF9A6873-1F22-5E17-B911-08E8F10CF253; **Location:** country: Russian Federation; countryCode: RU; stateProvince: Khanty-Mansiyskiy Avtonomnyy Okrug; county: Khanty-Mansiyskiy Rayon; locality: Mukhrino field station of YSU, 20 km SW from Khanty-Mansiysk; decimalLatitude: 60.891781; decimalLongitude: 68.684251; **Identification:** identifiedBy: Filippova, Nina|Zvyagina, Elena; dateIdentified: 2023-02-28; identificationRemarks: Identification based on morphological and molecular characters; **Event:** eventDate: 2022-08-19; habitat: Raised Sphagnum bog

#### Mycena pura

(Pers.) P.Kumm.

81C45058-FAE0-5984-A8D7-592B317DD99C

##### Materials

- **Type status:** Other material. **Occurrence:** recordedBy: Filippova, Nina; occurrenceID: 467E3637-37BD-5AD7-9F50-BBAC1AFA34AC; **Location:** country: Russian Federation; countryCode: RU; county: Khanty-Mansiyskiy Rayon; locality: Mukhrino field station of YSU, 20 km SW from Khanty-Mansiysk; decimalLatitude: 60.891781; decimalLongitude: 68.684251; **Identification:** identifiedBy: Filippova, Nina; identificationRemarks: Identification based on observation, no collections were made; **Event:** eventDate: 2022-08-19; habitat: Raised Sphagnum bog

#### Mycena cf. aff. megaspora

(Pers.) P.Kumm.

88911350-B8A2-51CF-84F6-411A5D80C139

##### Materials

- **Type status:** Other material. **Occurrence:** catalogNumber: YSU-F-04086; recordedBy: Filippova, Nina; associatedSequences: OP866189; occurrenceID: 2B03CF15-9F68-525C-A756-F6F3B824A5AC; **Location:** country: Russian Federation; countryCode: RU; stateProvince: Khanty-Mansiyskiy Avtonomnyy Okrug; county: Khanty-Mansiyskiy Rayon; locality: Mukhrino field station of YSU, 20 km SW from Khanty-Mansiysk; decimalLatitude: 60.892022; decimalLongitude: 68.691502; **Identification:** identifiedBy: Filippova, Nina|Zvyagina, Elena; dateIdentified: 2023-02-28; identificationRemarks: Identification based on morphological and molecular characters; **Event:** eventDate: 2012-09-09; habitat: Pine - dwarfshrubs - sphagnum ombrotrophic bog

#### Bogbodia uda

(Pers.) Redhead

2FC0D985-E8B5-5BAB-BF44-E6EB8D291B15

##### Materials

- **Type status:** Other material. **Occurrence:** catalogNumber: YSU-F-07317; recordedBy: Filippova, Nina; associatedSequences: OP866217; occurrenceID: AD803EAD-70DF-50C1-833B-E42C42A58543; **Location:** country: Russian Federation; countryCode: RU; stateProvince: Khanty-Mansiyskiy Avtonomnyy Okrug; county: Khanty-Mansiyskiy Rayon; locality: Mukhrino field station of YSU, 20 km SW from Khanty-Mansiysk; decimalLatitude: 60.891781; decimalLongitude: 68.684251; **Identification:** identifiedBy: Filippova, Nina|Zvyagina, Elena; dateIdentified: 2023-02-28; identificationRemarks: Identification based on morphological and molecular characters; **Event:** eventDate: 2016-09-05; habitat: Graminoid-Sphagnum ombrotrophic hollow
- **Type status:** Other material. **Occurrence:** catalogNumber: YSU-F-10527; recordedBy: Filippova, Nina; associatedSequences: OP866234; occurrenceID: 313A3813-D2EE-5FC5-9C65-4031F1AEBCBA; **Location:** country: Russian Federation; countryCode: RU; stateProvince: Khanty-Mansiyskiy Avtonomnyy Okrug; county: Khanty-Mansiyskiy Rayon; locality: Khanty-Mansiysk town vicinity; decimalLatitude: 60.891900; decimalLongitude: 68.682260; **Identification:** identifiedBy: Filippova, Nina|Zvyagina, Elena; dateIdentified: 2023-02-28; identificationRemarks: Identification based on morphological and molecular characters; **Event:** eventDate: 2020-09-08; habitat: Raised Sphagnum bog

#### Omphaliaster sp.

8D9CB004-3291-5F97-849B-3330F9B49C77

##### Materials

- **Type status:** Other material. **Occurrence:** catalogNumber: YSU-F-04122; recordedBy: Filippova, Nina; associatedSequences: OQ396705; occurrenceID: 5B297732-D6ED-5F14-95F8-868070ACA234; **Location:** country: Russian Federation; countryCode: RU; stateProvince: Khanty-Mansiyskiy Avtonomnyy Okrug; county: Khanty-Mansiyskiy Rayon; locality: Chistoe bog, 20 km E from Khanty-Mansiysk; decimalLatitude: 61.054422; decimalLongitude: 69.456725; **Identification:** identifiedBy: Filippova, Nina|Zvyagina, Elena; dateIdentified: 2023-02-28; identificationRemarks: Identification based on morphological and molecular characters; **Event:** eventDate: 2012-09-17; habitat: Pine - dwarfshrubs - sphagnum hummock (patterned ombrotrophic bog)

#### Psathyrella sphagnicola

(Maire) J.Favre

BE4E110B-42BB-562B-9F70-3F890B704069

##### Materials

- **Type status:** Other material. **Occurrence:** catalogNumber: YSU-F-04433; recordedBy: Filippova, Nina; associatedSequences: OP866200; occurrenceID: 5747BC2B-27EB-5C96-89AE-2F9EF1046008; **Location:** country: Russian Federation; countryCode: RU; stateProvince: Khanty-Mansiyskiy Avtonomnyy Okrug; county: Khanty-Mansiyskiy Rayon; locality: Mukhrino field station of YSU, 20 km SW from Khanty-Mansiysk; decimalLatitude: 60.892632; decimalLongitude: 68.677156; **Identification:** identifiedBy: Filippova, Nina|Zvyagina, Elena; dateIdentified: 2023-02-28; identificationRemarks: Identification based on morphological and molecular characters; **Event:** eventDate: 2013-09-18; habitat: Graminoid - sphagnum hollow (patterned ridge - hollow bog)

#### Pseudoplectania episphagnum

(J. Favre) M. Carbone, Agnello & P. Alvarado

1F634223-9C79-5349-B80F-97BFBE394D62

##### Materials

- **Type status:** Other material. **Occurrence:** catalogNumber: YSU-F-07716; recordedBy: Filippova, Nina; occurrenceID: C99AD73C-E065-5506-BD4D-BD2552D8761F; **Location:** country: Russian Federation; countryCode: RU; county: Khanty-Mansiyskiy Rayon; locality: Mukhrino field station of YSU, 20 km SW from Khanty-Mansiysk; decimalLatitude: 60.891781; decimalLongitude: 68.684251; **Event:** eventDate: 2017-06-03; habitat: Pine-dwarfshrubs-S.fuscum ombrotrophic bog

#### Pseudoplectania lignicola

Glejdura, V.Kučera, Lizoň & Kunca

EC99E55F-A1EE-58D0-A787-BAADBC3805FE

##### Materials

- **Type status:** Other material. **Occurrence:** catalogNumber: YSU-F-07713; recordedBy: Filippova, Nina; associatedSequences: OQ396706; occurrenceID: C0E1A760-ED3B-5FF9-AC8C-9DE4B33EA46B; **Location:** country: Russian Federation; countryCode: RU; stateProvince: Khanty-Mansiyskiy Avtonomnyy Okrug; county: Khanty-Mansiyskiy Rayon; locality: Mukhrino field station of YSU, 20 km SW from Khanty-Mansiysk; decimalLatitude: 60.891781; decimalLongitude: 68.684251; **Identification:** identifiedBy: Filippova, Nina|Zvyagina, Elena; dateIdentified: 2023-02-28; identificationRemarks: Identification based on morphological and molecular characters; **Event:** eventDate: 2017-06-03; habitat: Pine-dwarfshrubs-S.fuscum ombrotrophic bog

#### Psilocybe turficola

J.Favre

088926E5-2E11-5F0C-A5CA-F50089AADBEF

##### Materials

- **Type status:** Other material. **Occurrence:** catalogNumber: YSU-F-03896; recordedBy: Filippova, Nina; associatedSequences: OP866183; occurrenceID: 93046516-E43A-55A9-9175-CE7CAE76AB19; **Location:** country: Russian Federation; countryCode: RU; stateProvince: Khanty-Mansiyskiy Avtonomnyy Okrug; county: Khanty-Mansiyskiy Rayon; locality: Chistoe bog, 20 km E from Khanty-Mansiysk; decimalLatitude: 61.053093; decimalLongitude: 69.448142; **Identification:** identifiedBy: Filippova, Nina|Zvyagina, Elena; dateIdentified: 2023-02-28; identificationRemarks: Identification based on morphological and molecular characters; **Event:** eventDate: 2012-08-29; habitat: Sedge - sphagnum oligo-mesotrophic hollow

#### Russula decolorans

(Fr.) Fr.

BA5DDF1F-24ED-539F-A7C6-FE00F3CF89A9

##### Materials

- **Type status:** Other material. **Occurrence:** catalogNumber: YSU-F-08371; recordedBy: Filippova, Nina; associatedSequences: OP866226; occurrenceID: 7F70CD2E-ECAE-579F-84AA-E44AB4B4999C; **Location:** country: Russian Federation; countryCode: RU; stateProvince: Khanty-Mansiyskiy Avtonomnyy Okrug; county: Khanty-Mansiyskiy Rayon; locality: Mukhrino field station of YSU, 20 km SW from Khanty-Mansiysk; decimalLatitude: 60.891781; decimalLongitude: 68.684251; **Identification:** identifiedBy: Filippova, Nina|Zvyagina, Elena; dateIdentified: 2023-02-28; identificationRemarks: Identification based on morphological and molecular characters; **Event:** eventDate: 2018-08-28; habitat: Treed Pine-dwarfshrubs-Sphagnum bog

#### Russula emetica

(Schaeff.) Pers.

205AAF73-D149-5F82-BFCD-94ECB98973FA

##### Materials

- **Type status:** Other material. **Occurrence:** catalogNumber: YSU-F-04107; recordedBy: Filippova, Nina; associatedSequences: OQ396704; occurrenceID: DD8F7BC5-49F2-5EA4-B7B9-7A3797F6BAF2; **Location:** country: Russian Federation; countryCode: RU; stateProvince: Khanty-Mansiyskiy Avtonomnyy Okrug; county: Khanty-Mansiyskiy Rayon; locality: Mukhrino field station of YSU, 20 km SW from Khanty-Mansiysk; decimalLatitude: 60.900413; decimalLongitude: 68.691845; **Identification:** identifiedBy: Filippova, Nina|Zvyagina, Elena; dateIdentified: 2023-02-28; identificationRemarks: Identification based on morphological and molecular characters; **Event:** eventDate: 2012-09-11; habitat: Pine - dwarfshrubs - sphagnum bog (close to forest)

#### Russula paludosa

Britzelm.

F457FA09-D3F4-5200-8B2F-CFA8652D2991

##### Materials

- **Type status:** Other material. **Occurrence:** catalogNumber: YSU-F-03868; recordedBy: Filippova, Nina; associatedSequences: OP866182; occurrenceID: E821F179-5D5E-57B9-BD01-7030F656645C; **Location:** country: Russian Federation; countryCode: RU; stateProvince: Khanty-Mansiyskiy Avtonomnyy Okrug; county: Khanty-Mansiyskiy Rayon; locality: Chistoe bog, 20 km E from Khanty-Mansiysk; decimalLatitude: 61.060051; decimalLongitude: 69.459472; **Identification:** identifiedBy: Filippova, Nina|Zvyagina, Elena; dateIdentified: 2023-02-28; identificationRemarks: Identification based on morphological and molecular characters; **Event:** eventDate: 2012-08-28; habitat: Pine - dwarfshrubs - sphagnum ombrotrophic bog
- **Type status:** Other material. **Occurrence:** catalogNumber: YSU-F-08507; recordedBy: Filippova, Nina; associatedSequences: OQ396708; occurrenceID: EB58A1EE-0620-5DE6-81B6-F5743EFCE27B; **Location:** country: Russian Federation; countryCode: RU; stateProvince: Tomskaya Oblast'; county: Tomskiy Rayon; locality: Orlovka village vicinity, Chernoye lake; decimalLatitude: 56.878320; decimalLongitude: 84.665770; **Identification:** identifiedBy: Filippova, Nina|Zvyagina, Elena; dateIdentified: 2023-02-28; identificationRemarks: Identification based on morphological and molecular characters; **Event:** eventDate: 2018-08-22; habitat: Raised Pine-dwarfshrubs-Sphagnum bog

#### Sagaranella tylicolor

(Fr.) V.Hofst., Clémençon, Moncalvo & Redhead

E452DEDE-4000-5A88-B0F0-169E5AB748C6

##### Materials

- **Type status:** Other material. **Occurrence:** catalogNumber: YSU-F-07834; recordedBy: Filippova, Nina; occurrenceID: 234CA784-46BC-5E4E-AEDE-0145B9ECB620; **Location:** country: Russian Federation; countryCode: RU; county: Khanty-Mansiyskiy Rayon; locality: Mukhrino field station of YSU, 20 km SW from Khanty-Mansiysk; decimalLatitude: 60.891781; decimalLongitude: 68.684251; **Identification:** identifiedBy: Filippova, Nina; dateIdentified: 2017-07-08; identificationRemarks: Identification based on morphological characters only; **Event:** eventDate: 2017-07-08; habitat: Pine-dwarfshrubs-S.fuscum ombrotrophic bog

#### Sphagnurus paluster

(Peck) Redhead & V.Hofst.

3DFEBF58-DB4A-5495-99CE-743D5191E3E2

##### Materials

- **Type status:** Other material. **Occurrence:** catalogNumber: YSU-F-12022; recordedBy: Filippova, Nina; associatedSequences: OP866239; occurrenceID: 551A6A83-DF4B-5F51-954A-0FD172C603DA; **Location:** country: Russian Federation; countryCode: RU; stateProvince: Khanty-Mansiyskiy Avtonomnyy Okrug; county: Khanty-Mansiyskiy Rayon; locality: Mukhrino field station of YSU, 20 km SW from Khanty-Mansiysk; decimalLatitude: 60.891781; decimalLongitude: 68.684251; **Identification:** identifiedBy: Filippova, Nina|Zvyagina, Elena; dateIdentified: 2023-02-28; identificationRemarks: Identification based on morphological and molecular characters; **Event:** eventDate: 2022-07-11; habitat: Raised Sphagnum bog
- **Type status:** Other material. **Occurrence:** catalogNumber: YSU-F-12124; recordedBy: Filippova, Nina|Rudykina, Elena; associatedSequences: OP866245; occurrenceID: D366F273-316C-59DB-9959-5E36046E21EF; **Location:** country: Russian Federation; countryCode: RU; stateProvince: Khanty-Mansiyskiy Avtonomnyy Okrug; county: Khanty-Mansiyskiy Rayon; locality: Mukhrino field station of YSU, 20 km SW from Khanty-Mansiysk; decimalLatitude: 60.891781; decimalLongitude: 68.684251; **Identification:** identifiedBy: Filippova, Nina|Zvyagina, Elena; dateIdentified: 2023-02-28; identificationRemarks: Identification based on morphological and molecular characters; **Event:** eventDate: 2022-08-04; habitat: Raised Sphagnum bog

#### Strobilurus stephanocystis

(Kühner & Romagn. ex Hora) Singer

9DD4F963-3361-50B5-97D6-12A9CFC73AC8

##### Materials

- **Type status:** Other material. **Occurrence:** recordedBy: Filippova, Nina; occurrenceID: 066287C6-F9A5-5A25-8729-C37D0889823A; **Location:** country: Russian Federation; countryCode: RU; county: Khanty-Mansiyskiy Rayon; locality: Mukhrino field station of YSU, 20 km SW from Khanty-Mansiysk; decimalLatitude: 60.891781; decimalLongitude: 68.684251; **Identification:** identifiedBy: Filippova, Nina; identificationRemarks: Identification based on observation, no collections were made; **Event:** eventDate: 2022-08-19; habitat: Raised Sphagnum bog

#### Suillus praetermissus

Zvyagina & Svetash.

00B04160-E78A-5AAC-8367-88ABFF357770

##### Materials

- **Type status:** Other material. **Occurrence:** catalogNumber: YSU-F-04382; recordedBy: Filippova, Nina; associatedSequences: OQ407682; occurrenceID: 0026892C-5BB6-5C3E-AC79-EEB4AF1744B2; **Location:** country: Russian Federation; countryCode: RU; stateProvince: Khanty-Mansiyskiy Avtonomnyy Okrug; county: Khanty-Mansiyskiy Rayon; locality: Mukhrino field station of YSU, 20 km SW from Khanty-Mansiysk; decimalLatitude: 60.892632; decimalLongitude: 68.677156; **Identification:** identifiedBy: Filippova, Nina|Zvyagina, Elena; dateIdentified: 2023-02-28; identificationRemarks: Identification based on morphological and molecular characters; **Event:** eventDate: 2013-08-22; habitat: Dwarfshrubs - sphagnum ombrotrophic bog

#### Suillus flavidus

(Fr.) J.Presl

CC29B372-473C-5EB2-BF40-F51249CCEA19

##### Materials

- **Type status:** Other material. **Occurrence:** catalogNumber: YSU-F-03981; recordedBy: Filippova, Nina; associatedSequences: OP866186; occurrenceID: BA89F6AB-FE9B-5F39-A14D-A9B1E5C7D40A; **Location:** country: Russian Federation; countryCode: RU; stateProvince: Khanty-Mansiyskiy Avtonomnyy Okrug; county: Khanty-Mansiyskiy Rayon; locality: Mukhrino field station of YSU, 20 km SW from Khanty-Mansiysk; decimalLatitude: 60.892773; decimalLongitude: 68.674893; **Identification:** identifiedBy: Filippova, Nina|Zvyagina, Elena; dateIdentified: 2023-02-28; identificationRemarks: Identification based on morphological and molecular characters; **Event:** eventDate: 2012-09-02; habitat: Pine - dwarfshrubs - sphagnum hummock (patterned ombrotrophic bog)

#### Suillus punctipes

(Peck) Singer

98D35DD3-915F-5E26-93FD-5A4B6EA7511C

##### Materials

- **Type status:** Other material. **Occurrence:** catalogNumber: YSU-F-10533; recordedBy: Filippova, Nina; associatedSequences: OQ406272; occurrenceID: 7E714B52-5FD6-5274-A3C8-ED3D67CFC54D; **Location:** country: Russian Federation; countryCode: RU; stateProvince: Khanty-Mansiyskiy Avtonomnyy Okrug; county: Khanty-Mansiyskiy Rayon; locality: Khanty-Mansiysk town vicinity; decimalLatitude: 60.891900; decimalLongitude: 68.682260; **Identification:** identifiedBy: Filippova, Nina|Zvyagina, Elena; dateIdentified: 2023-02-28; identificationRemarks: Identification based on morphological and molecular characters; **Event:** eventDate: 2020-09-08; habitat: Raised Sphagnum bog

#### Thelephora terrestris

Ehrh.

5DBE2BF4-7F07-5523-B66A-03D587A587FD

##### Materials

- **Type status:** Other material. **Occurrence:** catalogNumber: YSU-F-04098; recordedBy: Filippova, Nina; occurrenceID: FFD804E6-BEAD-5355-8F8F-6AF842475E33; **Location:** country: Russian Federation; countryCode: RU; county: Khanty-Mansiyskiy Rayon; locality: Mukhrino field station of YSU, 20 km SW from Khanty-Mansiysk; decimalLatitude: 60.892022; decimalLongitude: 68.691502; **Identification:** identifiedBy: Filippova, Nina; dateIdentified: 2023-02-28; identificationRemarks: Identification based on morphological characters only; **Event:** eventDate: 2012-09-09; habitat: Pine - dwarfshrubs - sphagnum ombrotrophic bog

#### Tubaria furfuracea

(Pers.) Gillet

C6B1DB55-3D83-5C81-AACC-358CC2B65363

##### Materials

- **Type status:** Other material. **Occurrence:** recordedBy: Filippova, Nina; occurrenceID: 3DE6220F-DC39-53B2-B388-2C07D071F9E4; **Location:** country: Russian Federation; countryCode: RU; county: Khanty-Mansiyskiy Rayon; locality: Mukhrino field station of YSU, 20 km SW from Khanty-Mansiysk; decimalLatitude: 60.891781; decimalLongitude: 68.684251; **Identification:** identifiedBy: Filippova, Nina; identificationRemarks: Identification based on observation, no collections were made; **Event:** eventDate: 2022-08-19; habitat: Raised Sphagnum bog

#### Xeromphalina sp.

19036A6B-4DD1-596E-A282-1A696B6BEB61

##### Materials

- **Type status:** Other material. **Occurrence:** catalogNumber: YSU-F-03795; recordedBy: Filippova, Nina; associatedSequences: OQ407679; occurrenceID: 45720CA3-6B5C-5344-A3FC-49487DA9D41F; **Location:** country: Russian Federation; countryCode: RU; stateProvince: Khanty-Mansiyskiy Avtonomnyy Okrug; county: Khanty-Mansiyskiy Rayon; locality: Chistoe bog, 20 km E from Khanty-Mansiysk; decimalLatitude: 61.057330; decimalLongitude: 69.462476; **Identification:** identifiedBy: Filippova, Nina|Zvyagina, Elena; dateIdentified: 2023-02-28; identificationRemarks: Identification based on morphological and molecular characters; **Event:** eventDate: 2012-08-21; habitat: Dwarfshrubs - sphagnum ombrotrophic bog
- **Type status:** Other material. **Occurrence:** catalogNumber: YSU-F-05025; recordedBy: Filippova, Nina; associatedSequences: OQ407678; occurrenceID: 701F4CCB-8605-5FF3-89EE-6C93526795E5; **Location:** country: Russian Federation; countryCode: RU; stateProvince: Khanty-Mansiyskiy Avtonomnyy Okrug; county: Khanty-Mansiyskiy Rayon; locality: Mukhrino field station of YSU, 20 km SW from Khanty-Mansiysk; decimalLatitude: 60.891980; decimalLongitude: 68.682430; **Identification:** identifiedBy: Filippova, Nina|Zvyagina, Elena; dateIdentified: 2023-02-28; identificationRemarks: Identification based on morphological and molecular characters; **Event:** eventDate: 2015-06-13; habitat: Pine - dwarfshrubs - S. fuscum ombrotrophic bog

#### Xeromphalina campanelloides

Redhead

2C97E24C-E0DA-5766-B25F-97D7724B40FC

##### Materials

- **Type status:** Other material. **Occurrence:** catalogNumber: YSU-F-04042; recordedBy: Filippova, Nina; associatedSequences: OP866188; occurrenceID: E204C94B-7D1F-56E1-ADDA-6815E28B3768; **Location:** country: Russian Federation; countryCode: RU; stateProvince: Khanty-Mansiyskiy Avtonomnyy Okrug; county: Khanty-Mansiyskiy Rayon; locality: Chistoe bog, 20 km E from Khanty-Mansiysk; decimalLatitude: 61.066591; decimalLongitude: 69.457326; **Identification:** identifiedBy: Filippova, Nina|Zvyagina, Elena; dateIdentified: 2023-02-28; identificationRemarks: Identification based on morphological and molecular characters; **Event:** eventDate: 2012-09-07; habitat: Pine - dwarfshrubs - sphagnum bog (close to forest)

#### Xeromphalina setulipes

Esteve-Rav. & G.Moreno

77CAA6A1-3915-5854-A83B-C2095A024A83

##### Materials

- **Type status:** Other material. **Occurrence:** catalogNumber: YSU-F-05833; recordedBy: Filippova, Nina; associatedSequences: OQ406267; occurrenceID: DC11A58C-AADC-5BE7-B7D9-9981178C6F85; **Location:** country: Russian Federation; countryCode: RU; stateProvince: Khanty-Mansiyskiy Avtonomnyy Okrug; county: Khanty-Mansiyskiy Rayon; locality: Mukhrino field station of YSU, 20 km SW from Khanty-Mansiysk; decimalLatitude: 60.891781; decimalLongitude: 68.684251; **Identification:** identifiedBy: Filippova, Nina|Zvyagina, Elena; dateIdentified: 2023-02-28; identificationRemarks: Identification based on morphological and molecular characters; **Event:** eventDate: 2015-08-08; habitat: Graminoid-Sphagnum hollow in ombrotrophic bog

## Analysis

### Taxonomic diversity

**Figure 2.**
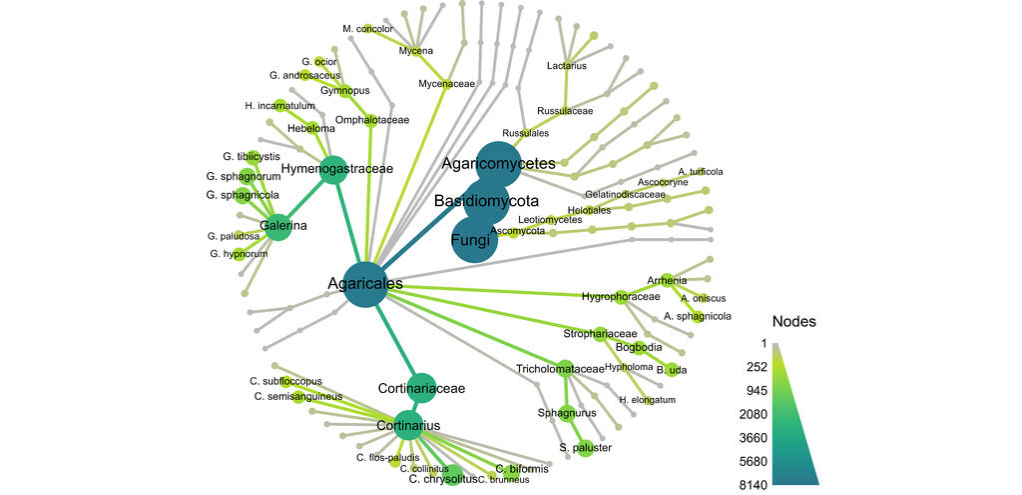
The taxonomical structure of the macromycetes community studied in the raised bog “Mukhrino” revealed by a complex approach. Node size and colour mark the number of occurrences.

Morphological identification, based on macro- and micro-morphological characters, revealed 79 species. Barcoding of a selected reference collection resulted in 95 species. Sixty-nine species were assigned by BLAST search reliably to taxonomically valid species, 14 taxa remain unassigned (‘molecular species’) and possibly represent under-described taxa, while 12 taxa remain unsequenced. Molecular identification added several cryptic species to the genera *Cortinarius*, *Galerina*, *Gymnopus* and *Russula*. Several sequences revealed potentially new species (*Arrhenia* sp., *Cortinarius* spp. (5), *Entoloma* sp., *Galerina* spp. (3), *Gymnopus* sp., *Gymnopilus* sp., *Mycena* sp., *Omphaliaster* sp. and *Xeromphalina* sp.), which were not assigned to any existing sequences using BLAST and require further study. Finally, the taxonomic diversity of macromycetes of the raised bog “Mukhrino” revealed by a complex approach is represented by 95 species from 33 genera, 23 families, seven orders, three classes and two phyla (Fig. 2, Suppl. material 1).

Eleven species are reported for the first time in Russia, based on a recently-published country-scale checklist of agaricoid and boletoid fungi (Bolshakov et al. 2021): *Cortinariusaurantiobasis*, *C.brunneotinctus*, *C.cruentiphyllus*, *C.davemallochii*, *C.kauffmanianus*, *C.lindstroemii*, *C.sphagnoravus*, *C.tenuifulvescens*, *Gymnopusjunquilleus*, *Xeromphalinacampanelloides* and *X.setulipes*.

### Estimates of species diversity

**Figure 3.**
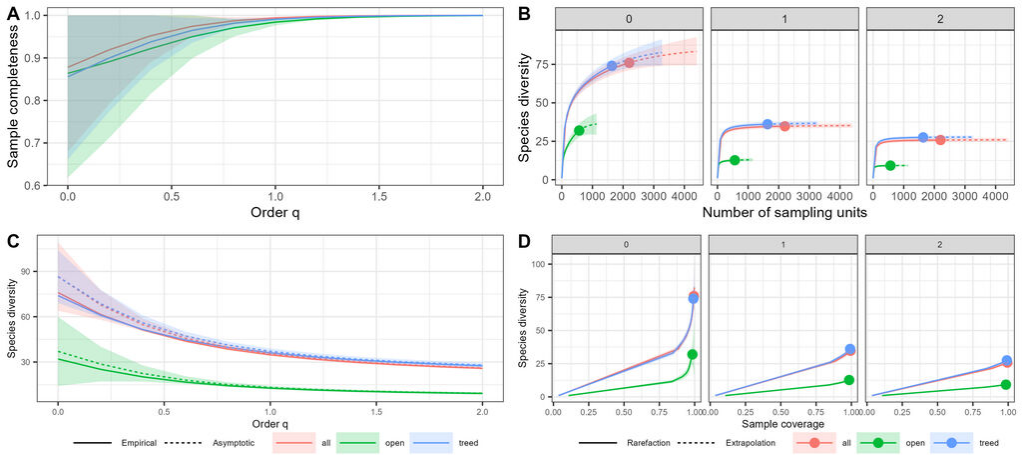
Species diversity estimates. **A** Estimated sample completeness curves as a function of order q between 0 and 2. **B** Sample-size-based rarefaction (solid lines) and extrapolation curves (dashed lines) up to double-sized samples. **C** Empirical diversity profiles (solid lines) and asymptotic estimates of diversity profiles (dotted lines). **D** Coverage-based rarefaction (solid lines) and extrapolation (dashed lines) curves up to the corresponding coverage value for double-sized samples (C_max_ = 99.7 %).

The results of the estimated sample completeness are shown in Fig. 3. Sample completeness was maximal for Simpson diversity (q = 2) and minimal for Shannon diversity (q = 1) (Fig. 3A). Sample-size-based rarefaction and extrapolation curves reached asymptote for the Simpson and Shannon diversity (q = 1, 2), demonstrating a slight growth in species richness (q = 0) (Fig. 3B). The asymptotic diversity estimations for the whole community and divided by two habitats is shown in Table 1. Under-estimated species diversity is at least 10.6 species for the raised bog community in total, 12.5 species for treed bogs and 5.1 species for open bog habitats. Comparison of the rarefaction and extrapolation sample-coverage curves confirms that the diversity of treed bogs is significantly higher than that of open bogs; this is true when comparing samples by all diversity orders (q = 0, 1 and 2) (Fig. 3C and D).

### Quantitative community structure

The total fruiting abundance, defined as the number of fruit-bodies of a particular species accumulated in the course of a year, varies by three orders of magnitude (from 1 to thousands of fruitbodies per hectare). Following the logarithmic abundance scale (Table 2), only six species are abundant, while 28 species are rare; others can be considered common and regular. The species abundance rank varies insignificantly between years, with the most abundant species always occupying the top positions amongst other species.

### Fruiting phenology

**Figure 4.**
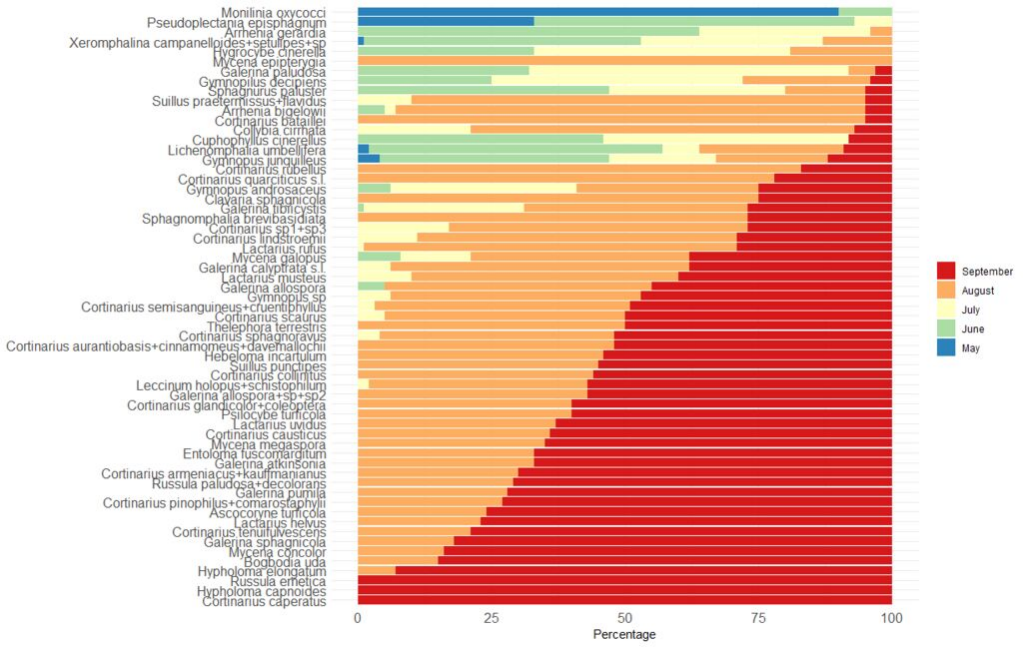
The relative number of fruit-bodies per species per month, mean for all years of observations (only species with over two observations are shown).

**Figure 5.**
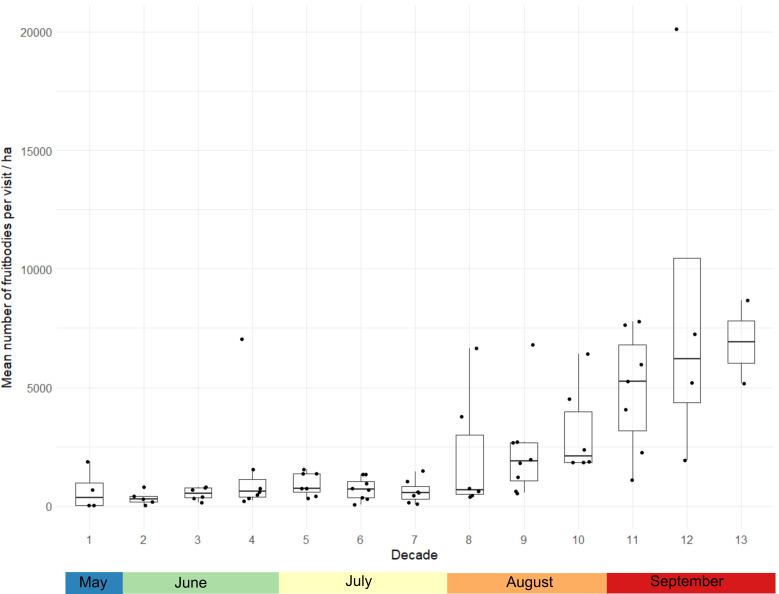
Seasonal dynamics of fruiting abundance, mean number of fruit-bodies per visit per hectare.

The fruiting of different species varies during the vegetation season. There are a few species fruiting in yearly summer (May-June), some species fruit in mid-summer (June – August); however, most species appear at the end of the season (August-September) (Fig. 4). The number of species per month varies from six in May to about 60 in September (Table 3). Fruiting abundance increases seasonally from spring to autumn by an order of magnitude, reaching its maximum in September (Fig. 5).

### Interannual dynamics

**Figure 6.**
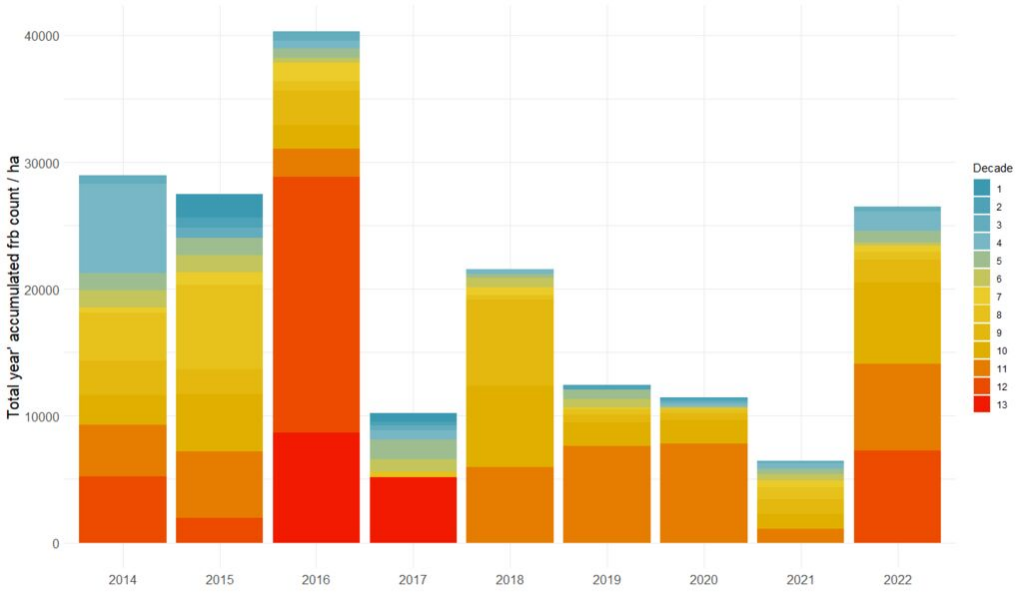
Fruiting dynamics between years, shown by the total number of accumulated fruit-bodies per year (colour showing decades).

The total abundance, or accumulated number of counted fruit-bodies, varies strongly between years: it reached the maximum of about 5000 frb/year/ha in 2016 and was the lowest in 2021 at 844 frb/year/ha (Fig. 6).

### Fungal community in relation to vegetation types

**Figure 7.**
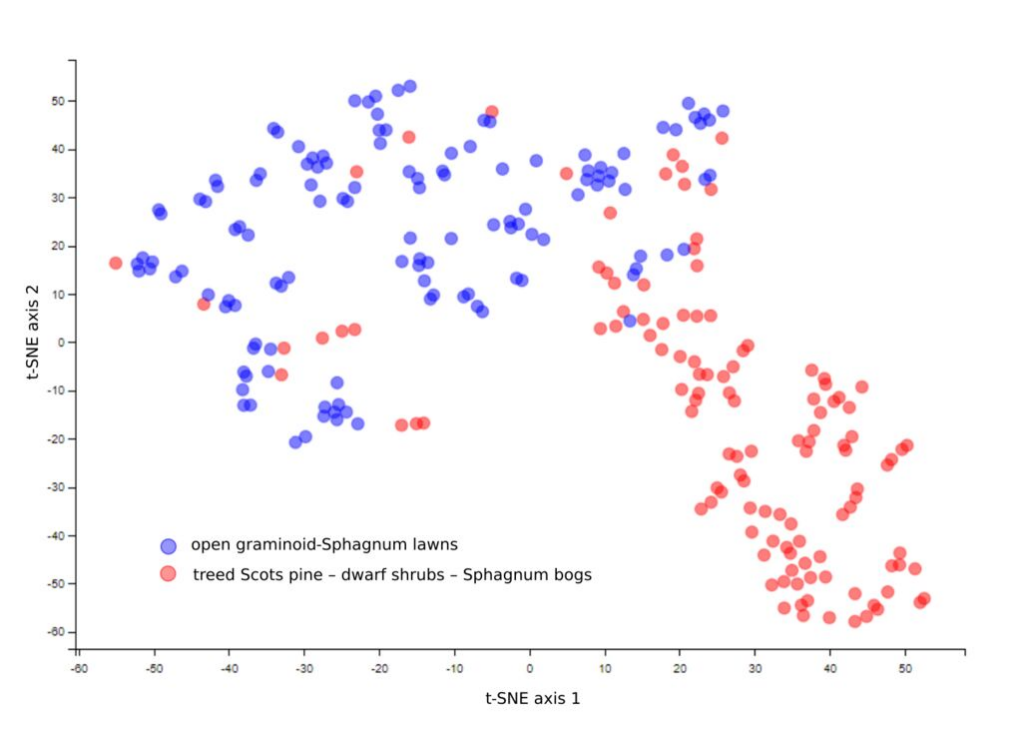
Arrangement of fungal community by two types of habitats (treed bogs and open *Sphagnum* lawns) using t-SNE ordination.

In order to study the influence of habitat on community composition of macromycetes, we created total species lists for each plot and arranged them according to habitat types and vegetation composition. Fig. 7 shows a sufficient distinction between the fungal composition of two habitats (treed bogs and open *Sphagnum* lawns).

## Discussion

### Taxonomic diversity

The previously-reported species diversity of fungi of peatlands globally (based on literature records) is estimated at about 600 species (Thormann and Rice 2007), including both micro- and macrofungi. This diversity is represented nearly equally by ascomycetes and basidiomycetes, with chytridiomycetes and zygomycetes being far less abundant. Based on the compilation created by Thormann and Rice (2007), we created an open-access literature-based global dataset of fungal records from peatlands (Filippova and Rudykina 2023). At the time of publication, the dataset included about 120 published works and about 5000 records of 1300 species of fungi from 15 countries. About a half of the published works used cultivation techniques (with a total of about 1000 records in the dataset), another half was derived from direct observation of fruiting structures of macromycetes (a total of about 4000 records in the dataset). The taxonomic structure of fungal diversity (after synonimisation using the GBIF species matching tool) includes three kingdoms (Fungi, Chromista, Protozoa), seven phyla, 26 classes, 80 orders, 212 families and about 1300 species. Macromycetes are represented by about 960 species, with only about 350 species of microfungi.

We used this dataset to compare our species list with the existing research of macromycetes in oligotrophic peatlands. Table 4 shows the summary of results by different authors (only the most complete studies conducted specifically in oligo- or mesotrophic raised bogs) by the total number of species revealed (about 20-70 species per study) and the proportion of shared species (up to 20%). The high number of unique species in each study may be explained by differences in the studied habitats or the broad scope of the habitats examined in each study (only a few works used the standardised plots located in explicitly-assigned vegetation types). The total number of species from all studies of macromycetes in oligotrophic peatlands is five times larger than our species list and the proportion of shared species is only 40% (28 species) from our species list. The unique species in our study are either species mostly restricted to our region overlooked by other researchers or potentially new taxa.

### Phenology

The phenology of fruiting of macromycetes in raised bog communities was studied by different authors (Lange 1948, Kotlaba 1953, Salo 1979, Saari and Salonen 1983). All authors agree about the seasonal development of fruiting, with the maximum fruiting abundance and number of species developing from August to October. We assumed a lack of data on long-term fruiting dynamics in peatlands in literature before this study. Several papers show a positive effect of peatland drainage on fruiting abundance (Chastukhin 1965, Veijalainen 1974, Kalamees 1982). Previous authors have shown the influence of a combination of factors (precipitation, soil or air humidity, soil temperature, soil heat flux and others) on fungal fruiting in general (Arnolds 1981, Kotilová-Kubičková et al. 1990, Straatsma et al. 2001, Pinna et al. 2010) and also registered global changes of fungal fruiting with climate change (Gange et al. 2007, Kauserud et al. 2008, Büntgen et al. 2013).

### Conservation initiatives

Macromycetes adapted to peatland ecosystems deserve special attention in conservation initiatives due to the ongoing degradation of these habitats on the global scale (Arnolds 2007). Species with low abundance, as well as species restricted to raised bog habitats, should be considered as targets in conservation programmes. We evaluated the list of species restricted to raised bog habitats according to their conservation status in Europe and found that many species were already included in conservation lists of many countries (Table 5). Understanding the ecology and population dynamics of these species is especially important in the light of their conservation.

## Supplementary Material

XML Treatment for Amanita porphyria

XML Treatment for Arrhenia bigelowii

XML Treatment for Arrhenia gerardiana

XML Treatment for Arrhenia philonotis

XML Treatment for Arrhenia sp.

XML Treatment for Ascocoryne turficola

XML Treatment for Auriscalpium vulgare

XML Treatment for Cantharellula umbonata

XML Treatment for Clavaria sphagnicola

XML Treatment for Collybia cirrhata

XML Treatment for Cortinarius armeniacus

XML Treatment for Cortinarius aurantiobasis

XML Treatment for Cortinarius bataillei

XML Treatment for Cortinarius brunneotinctus

XML Treatment for Thaxterogaster causticus

XML Treatment for Cortinarius cinnamomeus

XML Treatment for Cortinarius coleoptera

XML Treatment for Cortinarius collinitus

XML Treatment for Thaxterogaster comarostaphylidis

XML Treatment for Cortinarius cruentiphyllus

XML Treatment for Cortinarius davemallochii

XML Treatment for Cortinarius glandicolor

XML Treatment for Cortinarius kauffmanianus

XML Treatment for Cortinarius lindstroemii

XML Treatment for Cortinarius malachius

XML Treatment for Thaxterogaster pinophilus

XML Treatment for Cortinarius quarciticus

XML Treatment for Cortinarius rubellus

XML Treatment for Cortinarius scaurus

XML Treatment for Cortinarius semisanguineus

XML Treatment for Cortinarius sphagnoravus

XML Treatment for Cortinarius suberi

XML Treatment for Cortinarius tenuifulvescens

XML Treatment for Cortinarius biformis

XML Treatment for Cortinarius venustus

XML Treatment for Cortinarius sp.

XML Treatment for Cortinarius sp. 1

XML Treatment for Cortinarius sp. 2

XML Treatment for Cortinarius sp. 3

XML Treatment for Cortinarius sp. 4

XML Treatment for Cuphophyllus cinerellus

XML Treatment for Entoloma sp.

XML Treatment for Entoloma fuscomarginatum

XML Treatment for Galerina allospora

XML Treatment for Galerina calyptrata

XML Treatment for Galerina paludosa

XML Treatment for Galerina pumila

XML Treatment for Galerina sphagnicola

XML Treatment for Galerina tibiicystis

XML Treatment for Galerina sp.

XML Treatment for Galerina sp. 1

XML Treatment for Galerina sp. 2

XML Treatment for Gymnopilus decipiens

XML Treatment for Gymnopilus sp.

XML Treatment for Gymnopus androsaceus

XML Treatment for Gymnopus dryophilus

XML Treatment for Gymnopus junquilleus

XML Treatment for Gymnopus sp.

XML Treatment for Gyroflexus brevibasidiatus

XML Treatment for Hebeloma incarnatulum

XML Treatment for Hypholoma capnoides

XML Treatment for Hypholoma elongatum

XML Treatment for Laccaria proxima

XML Treatment for Lactarius helvus

XML Treatment for Lactarius musteus

XML Treatment for Lactarius rufus

XML Treatment for Lactarius uvidus

XML Treatment for Lactarius vietus

XML Treatment for Leccinum holopus

XML Treatment for Leccinum schistophilum

XML Treatment for Lichenomphalia umbellifera

XML Treatment for Mycena sp.

XML Treatment for Mycena galopus

XML Treatment for Mycena megaspora

XML Treatment for Mycena pura

XML Treatment for Mycena cf. aff. megaspora

XML Treatment for Bogbodia uda

XML Treatment for Omphaliaster sp.

XML Treatment for Psathyrella sphagnicola

XML Treatment for Pseudoplectania episphagnum

XML Treatment for Pseudoplectania lignicola

XML Treatment for Psilocybe turficola

XML Treatment for Russula decolorans

XML Treatment for Russula emetica

XML Treatment for Russula paludosa

XML Treatment for Sagaranella tylicolor

XML Treatment for Sphagnurus paluster

XML Treatment for Strobilurus stephanocystis

XML Treatment for Suillus praetermissus

XML Treatment for Suillus flavidus

XML Treatment for Suillus punctipes

XML Treatment for Thelephora terrestris

XML Treatment for Tubaria furfuracea

XML Treatment for Xeromphalina sp.

XML Treatment for Xeromphalina campanelloides

XML Treatment for Xeromphalina setulipes

F3E8A274-3A77-5B6A-88DA-07BBF1B3461D 10.3897/BDJ.11.e105111.suppl1 Supplementary material 1 Sequenced specimens of macromycetes accumulated during the monitoring period with corresponding morphology- and molecular-based identifications Data type sequences Brief description The table contains information about sequenced specimens with corresponding fields: Catalogue Number, GenBank number, Region, BOLD ID, Morphologically-defined species, DNA-defined species, UNITE SH, Closest sequence in GenBank, Closest Type specimen in GenBank, Percentage identity to the closest sequence in GenBank, Closest GenBank sequence citation. File: oo_918329.csv https://binary.pensoft.net/file/918329 Filippova N.V., Zvyagina E.A.

819E156C-9502-5158-AA72-96C1559C4171 10.3897/BDJ.11.e105111.suppl2 Supplementary material 2 Plot-based counts of macrofungi in the raised bog "Mukhrino" Data type occurrences Brief description The table contains the results of plot-based counts for 9 years on permanent plots located in the raised bog "Mukhrino", with corresponding fields: eventID (count event ID), parentEventID (plot ID), habitat (vegetation), decimalLatitude, decimalLongitude, minimumElevationInMeters, samplingProtocol, sampleSizeValue, sampleSizeUnit, eventDate, year, eventDate, country, countryCode, stateProvince, municipality, locality, geodeticDatum, coordinateUncertaintyInMeters, basisOfRecord, occurrenceID, scientificName, genus, organismQuantity, organismQuantityType, kingdom.
This table is published as a GBIF Sampling Event dataset and will be updated there (https://www.gbif.org/dataset/acd76923-54da-4799-b4b0-cfe585c2c0b8). File: oo_835555.csv https://binary.pensoft.net/file/835555 Filppova N.V., Zvyagina E.A., Rudykina E.A., Dobrynina A.S.

## Figures and Tables

**Table 1.** Asymptotic diversity estimation for species richness (q = 0), Shannon diversity (q = 1) and Simpson diversity (q = 3) for the community of macromycetes in peatlands in total (all data) and divided by two habitats (treed bogs, open bogs).

**Assemblage**	**Type**	**q = 0**	**q = 1**	**q = 2**
All data	Empirical	76.0	34.8	25.8
All data	Asymptotic	86.6	35.5	26.1
All data	Undetected	10.6	0.7	0.3
Treed bogs	Empirical	74.0	36.1	27.5
Treed bogs	Asymptotic	86.5	37.1	28.0
Treed bogs	Undetected	12.5	1.0	0.5
Open bogs	Empirical	32.0	12.7	9.2
Open bogs	Asymptotic	37.1	13.2	9.3
Open bogs	Undetected	5.1	0.5	0.1

**Table 2.** Fruiting abundance classes, using logarithmic abundance scale, total number of fruit-bodies per 1 ha over a year (mean number between all years).

**Abundance scale**	**Number of species**	**Species list**
Abundant (> 1000 frb/ha)	6	*Galerinasphagnicola*, *Cortinariusaurantiobasis*+*cinnamomeus*+*davemallochii*, *Sphagnuruspaluster*, *Galerinaallospora*+sp+sp2, *Bogbodiauda*, *Cortinariusarmeniacus*+*kauffmanianus*
Common (100 - 1000 frb/ha)	18	*Galerinatibiicystis*, *Galerinacalyptrata* s.l., *Gymnopusandrosaceus*, *Hebelomaincarnatulum*, *Cortinariussemisanguineus*+*cruentiphyllus*, *Cortinariustenuifulvescens*, *Arrheniagerardiana*, *Cortinariussphagnoravus*, *Moniliniaoxycocci*, *Hypholomaelongatum*, *Mycenaconcolor*, *Arrheniabigelowii*, *Pseudoplectaniaepisphagnum*, *Lactariusrufus*, *Galerinapaludosa*, *Cortinariusglandicolor*/Coleoptera, *Gymnopusjunquilleus*, *Ascocoryneturficola*
Regular (10-100 frb/ha)	21	*Cortinariuslindstroemii*, *Cortinariuscollinitus*, *Collybiacirrhata*, *Sphagnomphaliabrevibasidiata*, *Lichenomphaliaumbellifera*, *Galerinaallospora*, *Gymnopilusdecipiens*, *Cortinariuscausticus*, *Galerinapumila*, *Mycenagalopus*, *Cortinariusquarciticus* s.l., *Cortinariusscaurus*, *Suilluspuctipes*, *Suilluspraetermissus*+*flavidus*, *Xeromphalinacampanelloides*+*setulipes*+sp., *Cortinarius* sp1+sp3, *Cortinariusbataillei*, *Gymnopus* sp., *Mycenamegaspora*, *Cuphophylluscinerellus*, *Lactariushelvus*
Rare (< 10 frb/ha)	28	*Cortinariuspinophilus*+*comarostaphylii*, *Lactariusmusteus*, *Russulaemetica*, *Lactariusuvidus*, *Hygrocybecinerella*, *Hypholomacapnoides*, *Leccinumholopus*+*schistophilum*, *Russulapaludosa*+*decolorans*, *Cortinariusrubellus*, *Psilocybeturficola*, *Clavariasphagnicola*, *Thelephoraterrestris*, *Cortinariuscaperatus*, *Entolomafuscomarginatum*, *Galerinaatkinsoniana*, *Mycenaepipterygia*, *Amanitaporphyria*, *Cantharellulaumbonata*, *Entolomafernandae*, *Gynnopilus* sp., *Laccariaproxima*, *Lyophyllumtylicolor*, *Omphaliaster* sp., *Tubariafurfuracea*, *Auriscalpiumvulgare*, *Mycenapura*, *Pseudoplectanialignicola*, *Strobilurusstephanocystis*

**Table 3.** Total number and the list of species registered by month, for all years of observations.

**Month**	**Number of species**	**Species list**
May	6	*Moniliniaoxycocci*, *Pseudoplectaniaepisphagnum*, *Gymnopusocior*, *Arrheniasphagnicola*, *Lichenomphaliaumbellifera*
June	17	*Sphagnuruspaluster*, *Arrheniasphagnicola*, *Gymnopusjunquilleus*, *Pseudoplectaniaepisphagnum*, *Arrheniabigelowii*, *Gymnopusandrosaceus*, *Moniliniaoxycocci*, *Galerinapaludosa*, *Galerinatibiicystis*, *Lichenomphaliaumbellifera*, *Xeromphalinacampanelloides*+*setulipes*+sp., *Galerinacalyptrata* s.l., *Galerinaallospora*+sp+sp2, *Mycenagalopus*, *Cuphophylluscinerellus*, *Pseudoplectanialignicola*, *Strobilurusstephanocystis*
July	31	*Sphagnuruspaluster*, *Galerinatibiicystis*, *Arrheniagerardiana*, *Gymnopusandrosaceus*, *Cortinariusaurantiobasis*+*cinnamomeus*+*davemallochii*, *Arrheniabigelowii*, *Galerinapaludosa*, *Galerinacalyptrata* s.l., *Gymnopusjunquilleus*, *Galerinaallospora*+sp.+sp., *Cortinariussemisanguineus*+*cruentiphyllus*, *Gymnopilusdecipiens*, *Xeromphalinacampanelloides*+*setulipes*+sp., *Sphagnomphaliabrevibasidiata*, *Cortinariuslindstroemii*, *Mycenagalopus*, *Cuphophylluscinerellus*, *Pseudoplectaniaepisphagnum*, *Lichenomphaliaumbellifera*, *Suilluspraetermissus*+*flavidus*, *Collybiacirrhata*, *Galerinasphagnicola*, *Cortinariusarmeniacus*+*kauffmanianus*, *Bogbodiauda*, *Hebelomaincarnatulum*, *Cortinariussphagnoravus*, *Lactariusrufus*, *Cortinariusscaurus*, *Gymnopus* sp., *Entolomafernandae*, *Lyophyllumtylicolor*
August	56	*Cortinariusaurantiobasis*+*cinnamomeus*+*davemallochii*, *Galerinaallospora*+sp+sp2, *Cortinariusarmeniacus*+*kauffmanianus*, *Galerinatibiicystis*, *Galerinacalyptrata* s.l., *Sphagnuruspaluster*, *Galerinasphagnicola*, *Hebelomaincarnatulum*, *Cortinariussemisanguineus*+*cruentiphyllus*, *Bogbodiauda*, *Cortinariussphagnoravus*, *Cortinariustenuifulvescens*, *Gymnopusandrosaceus*, *Arrheniagerardiana*, *Gymnopusjunquilleus*, *Cortinariusglandicolor*+*coleoptera*, *Sphagnomphaliabrevibasidiata*, *Cortinariuslindstroemii*, *Cortinariuscollinitus*, *Arrheniabigelowii*, *Lactariusrufus*, *Mycenaconcolor*, *Suilluspraetermissus*+*flavidus*, *Ascocoryneturficola*, *Cortinariusbataillei*, *Gymnopilusdecipiens*, *Galerinaallospora*, *Collybiacirrhata*, *Suilluspuctipes*, *Cortinariuscausticus*, *Cortinarius* sp1+sp3, *Galerinapaludosa*, *Gymnopus* sp., *Mycenagalopus*, *Cortinariusscaurus*, *Lichenomphaliaumbellifera*, *Hypholomaelongatum*, *Lactariusmusteus*, *Cortinariusquarciticus* s.l., *Cortinariusrubellus*, *Xeromphalinacampanelloides*+*setulipes*+sp., *Galerinapumila*, *Russulapaludosa*, *Clavariasphagnicola*, *Lactariusuvidus*, *Pseudoplectaniaepisphagnum*, *Cortinariuspinophilus*+*comarostaphylii*, *Lactariushelvus*, *Leccinumholopus*+*schistophilum*, *Cantharellulaumbonata*, *Galerinaatkinsoniana*, *Psilocybeturficola*, *Thelephoraterrestris*, *Amanitaporphyria*, *Mycenaepipterygia*, *Mycenapura*
September	57	*Cortinariusaurantiobasis*+*cinnamomeus*+*davemallochii*, *Galerinasphagnicola*, *Cortinariusarmeniacus*+*kauffmanianus*, *Galerinaallospora*+sp+sp2, *Bogbodiauda*, *Cortinariustenuifulvescens*, *Hebelomaincarnatulum*, *Cortinariussemisanguineus*+*cruentiphyllus*, *Galerinacalyptrata* s.l., *Galerinatibiicystis*, *Cortinariussphagnoravus*, *Mycenaconcolor*, *Hypholomaelongatum*, *Gymnopusandrosaceus*, *Sphagnuruspaluster*, *Cortinariusglandicolor*+*coleoptera*, *Ascocoryneturficola*, *Cortinariuscollinitus*, *Gymnopusjunquilleus*, *Lactariusrufus*, *Galerinaallospora*, *Cortinariuscausticus*, *Sphagnomphaliabrevibasidiata*, *Cortinariuslindstroemii*, *Galerinapumila*, *Cortinariusscaurus*, *Suilluspunctipes*, *Cortinariuspinophilus*+*comarostaphylii*, *Arrheniabigelowii*, *Gymnopus* sp., *Mycenagalopus*, *Russulaemetica*, *Collybiacirrhata*, *Galerinapaludosa*, *Lactariusmusteus*, *Russulapaludosa*+*decolorans*, *Lactariushelvus*, *Leccinumholopus*+*schistophillum*, *Hypholomacapnoides*, *Mycenamegaspora*, *Entolomafuscomarginatum*, *Omphaliaster* sp., *Suilluspraetermissus*+*flavidus*, *Cortinariusbataillei*, *Gymnopilusdecipiens*, *Lichenomphaliaumbellifera*, *Clavariasphagnicola*, *Lactariusuvidus*, *Cantharellulaumbonata*, *Galerinaatkinsoniana*, *Psilocybeturficola*, *Thelephoraterrestris*, *Cuphophylluscinerellus*, *Cortinariuscaperatus*, *Gynnopilus* sp., *Laccariaproxima*, *Tubariafurfuracea*

**Table 4.** Comparison of species lists from the studies of macromycetes in raised bogs globally: the lower part shows the absolute numbers of shared species; the upper part (itallic bold) shows the percentage of shared species; the intersection provides the total number of species in each publication.

	1. PS	2. Fl87	3. Fr97	4. Hol97	5. Km 82	6. KmR 82	7. Lan48	8. Ł07	9. Oh74	10. Rob04	11. Sal79	12. Sl04	13. St11	14. Tan00	15. Ch65	Totally
1. Present study	**95**	***11***	***7***	***9***	***10***	***6***	***11***	***9***	***10***	***5***	***13***	***11***	***16***	***1***	***6***	***7***
2. Flesinska 1987	12	**46**	***17***	***6***	***12***	***15***	***15***	***14***	***11***	***3***	***14***	***14***	***18***	***5***	***5***	***8***
3. Friedrich 1997	6	11	**20**	***10***	***7***	***15***	***14***	***13***	***21***	***3***	***12***	***12***	***18***	***1***	***8***	***4***
4. Holec 1997	9	5	5	**32**	***11***	***22***	***11***	***9***	***14***	***3***	***11***	***13***	***16***	***2***	***8***	***1***
5. Kalamees 1982	12	12	5	9	**53**	***17***	***11***	***11***	***11***	***4***	***14***	***9***	***15***	***0***	***1***	***9***
6. Kalamees and Raitviir 1982	6	7	3	7	14	**31**	***8***	***7***	***12***	***3***	***8***	***7***	***11***	***1***	***4***	***4***
7. Lange 1948	13	15	10	9	12	7	**53**	***13***	***14***	***8***	***16***	***18***	***18***	***4***	***9***	***10***
8. Łuszczyński 2007	11	14	10	8	12	6	14	**55**	***11***	***5***	***14***	***17***	***20***	***7***	***8***	***10***
9. Ohenoja 1974	9	7	8	7	8	6	10	8	**19**	***5***	***17***	***11***	***14***	***3***	***6***	***5***
10. Roberts et al. 2004	7	3	3	3	5	3	10	6	4	**66**	***6***	***6***	***5***	***3***	***0***	***6***
11. Salo 1979	19	17	11	12	18	8	20	18	16	9	**74**	***17***	***20***	***6***	***6***	***15***
12. Slusarczyk 2004	15	15	10	12	11	7	21	20	9	8	23	**64**	***23***	***3***	***10***	***3***
13. Stasinska 2011	20	19	14	14	16	10	20	22	11	6	26	28	**57**	***4***	***11***	***12***
14. Tanase 2000	1	5	1	2	0	1	4	8	2	4	8	4	4	**57**	***0***	***4***
15. Chastukhin 1965	5	3	3	4	1	2	6	6	2	0	5	8	8	0	**17**	***3***
Total species list	28	31	17	4	35	17	38	37	18	25	57	11	48	15	11	**388**

**Table 5.** Conservation status of species specialised to raised bog habitats, in Red Data books of several European countries (http://www.eccf.eu/activities-en.ehtml)

**N**	**Species**	**Protection status in different countries**
1	*Arrheniagerardiana*	Austria (2), Czech Republic (EN), France (4), Germany (endangered), Latvia (LC), Netherlands (EN), Norway (LC), Poland (V), Serbia (DD), Switzerland (EN)
2	*Ascocoryneturficola*	Norway (DD), Sweden (NT)
3	*Clavariasphagnicola*	France (1), Germany (critically endangered)
4	*Cortinariushuronensis*	Czech Rupublic (DD), Italy (X), Latvia (LC), Netherlands (SU)
5	*Entolomafuscomarginatum*	Denmark (EN), Netherlands (SU), Norway (NE)
6	*Galerinasphagnicola*	Austria (2)
7	*Gymnopilusfulgens*	Austria (2), Czech Republic (RE), Denmark (E 1997), Finland (DD), France (5), Germany (very rare/potentially endangered), Latvia (DD), Netherlands (VU), Norway (NE), Russia (3)
8	*Lactariusmusteus*	Austria (2), Czech Republic (EN), Denmark (VU), Germany (endangered), Great Britain (NT), Latvia (VU), Norway (LC), Sweden (NT), Switzerland (EN)
9	*Lichenomphaliaumbellifera*	France (5)
10	*Omphaliasterborealis*	Germany (critically endangered), Norway (LC)
11	*Psathyrellasphagnicola*	Czech Republic (CR), Denmark (RE 1997), France (2), Germany (endangered), Latvia (DD), Norway (NE), Switzerland (ED)
12	*Pseudoplectaniaepisphagnum*	Czech Republic (RE), Germany (critically endangered), Norway (NT)
13	*Psilocybeturficola*	Denmark (EN), France (2), Netherlands (SU), Poland (E)
14	*Sphagnomphaliabrevibasidiata*	France (1), Norway (NE), Switzerland (CR)
15	*Suillussibiricus*	Austria (3), Bulgaria (V), Czech Republic (CR), Estonia (CR), Germany (endangered), Poland (E), Slovakia (VU), Switzerland (VU)
